# Myosin-5 varies its steps along the irregular F-actin track

**DOI:** 10.1101/2023.07.16.549178

**Published:** 2023-07-16

**Authors:** Adam Fineberg, Yasuharu Takagi, Kavitha Thirumurugan, Joanna Andrecka, Neil Billington, Gavin Young, Daniel Cole, Stan A. Burgess, Alistair P. Curd, John A. Hammer, James R. Sellers, Philipp Kukura, Peter J. Knight

**Affiliations:** 1 Physical and Theoretical Chemistry Laboratory, Department of Chemistry, University of Oxford, Oxford OX1 3QZ, U.K.; 2 Laboratory of Single Molecule Biophysics, NHLBI, National Institutes of Health, Bethesda, Maryland 20892, U.S.A.; 3 Laboratory of Molecular Physiology, NHLBI, National Institutes of Health, Bethesda, Maryland 20892, U.S.A.; 4 Astbury Centre for Structural Molecular Biology, and Institute of Molecular and Cellular Biology, University of Leeds, Leeds, LS2 9JT, U.K.; 5 Cell and Developmental Biology Center, NHLBI, National Institutes of Health, Bethesda, MD 20892, U.S.A.; 6 The Kavli Institute for Nanoscience Discovery, Dorothy Crowfoot Hodgkin Building, University of Oxford, South Parks Rd, Oxford OX1 3QU, U.K.; 7 Present address: Structural Biology Lab, Pearl Research Park, SBST, Vellore Institute of Technology, Vellore-632 014, India; 8 Present address: Human Technopole, Viale Rita Levi-Montalcini 1, 20157, Milan, Italy; 9 Present address: Department of Biochemistry and Molecular Medicine, West Virginia University, Morgantown, WV, U.S.A.; 10 Present address: Refeyn Ltd., Unit 9, Trade City, Sandy Ln W, Littlemore, Oxford OX4 6FF, U.K.

**Keywords:** Myosin-5, Actin Filament, Cumulative Angular Disorder (CAD), Interferometric Scattering (iSCAT) Microscopy, Cryogenic Electron Microscopy (cryoEM), Negative-stain Electron Microscopy (nsEM), Single Molecule Biophysics

## Abstract

Molecular motors employ chemical energy to generate unidirectional mechanical output against a track. By contrast to the majority of macroscopic machines, they need to navigate a chaotic cellular environment, potential disorder in the track and Brownian motion. Nevertheless, decades of nanometer-precise optical studies suggest that myosin-5a, one of the prototypical molecular motors, takes uniform steps spanning 13 subunits (36 nm) along its F-actin track. Here, we use high-resolution interferometric scattering (iSCAT) microscopy to reveal that myosin takes strides spanning 22 to 34 actin subunits, despite walking straight along the helical actin filament. We show that cumulative angular disorder in F-actin accounts for the observed proportion of each stride length, akin to crossing a river on variably-spaced stepping stones. Electron microscopy revealed the structure of the stepping molecule. Our results indicate that both motor and track are soft materials that can adapt to function in complex cellular conditions.

## Introduction

**Figure 1.**
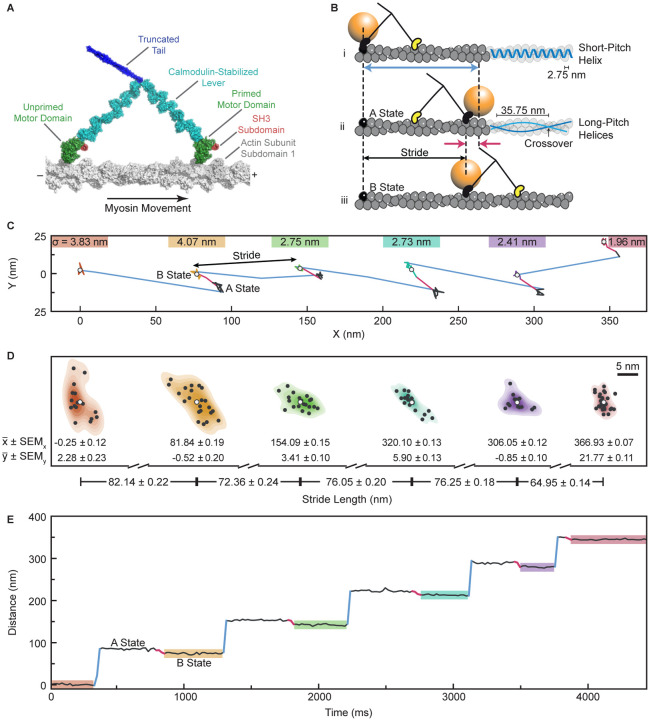
Trajectory along F-actin of a myosin-5a motor labelled with a gold bead. (A) Diagram of myosin-5a, truncated after the proximal coiled coil. (B) Schematic of two consecutive myosin steps, depicting first, the labelled head movement (blue arrow; B-state to A-state) and second, the AB transition of the bead (red arrows) accompanying the unlabeled head movement and allowing measurement of the labelled head stride (B-state to B-state). Also shown at right is actin’s short-pitch helix (top filament; bar indicates separation of successive subunits along that helix) and two long-pitch helices (middle filament; bar indicates spacing of successive crossovers). (C) The XY trace of a typical run along F-actin of a gold labelled myosin-5a molecule, highlighting labelled head strides (blue) and AB transitions (red). We report stride lengths as the Euclidean distance between the mean x and y values of consecutive B states. Localization precisions (σ), defined as the root sum of squares of x and y standard deviations of stationary states, marked above each B state. (D) The 2D kernel density estimates of bead positions highlighted in (C) with black points at each localization and white points at the mean values ($\bar{\text{x}}$, $\bar{\text{y}}$). Distances between the means demonstrate our high precision of stride length measurements. Uncertainty on mean particle position is standard error on mean (SEM_x_, SEM_y_), propagated to the error on stride sizes. (E) Distance along the filament versus Time trace of the XY plot in (C), again, highlighting labelled head movements (blue) and AB transitions (red).

Myosin-5a is a vital cellular motor^1–3^. It transports cargoes by converting chemical energy from ATP hydrolysis into unidirectional, processive motion along an actin filament. It has two heads, each comprising a motor domain connected to a coiled-coil tail via a long, *α*-helical lever that extends from the converter subdomain of the motor^4–6^ (Figure 1A). The molecule terminates in a dimeric domain that binds cargo. The ~22 nm lever helix is stabilized by six calmodulin-family light chains^6,7^. The mechanochemical mechanism is tightly coupled to ATP hydrolysis and the kinetics are limited by ADP release^8–10^. ATP binding induces a change in motor conformation that allows catalysis of ATP hydrolysis and is coupled to a swing of the lever into a ‘primed’ (also known as pre-powerstroke) orientation. Stereospecific binding of this primed motor to F-actin catalyzes release of phosphate and ADP, coupled to swinging of the primed lever (the powerstroke) back to its unprimed (post-powerstroke) angle, thus causing movement of attached cargoes along the actin^5–7,11–14^. Binding of another ATP causes rapid dissociation from actin and the cycle repeats. The ATPase kinetics of the two-headed myosin-5a on actin are such that both motor domains are bound to actin most of the time, with ADP in the active site^10^. In the two head-attached state, ADP release from the lead head is markedly suppressed (‘gated’) allowing the trail head to release its ADP and then rapidly bind ATP resulting in dissociation of this head from actin^9,10,15,16^. The attached head then undergoes the powerstroke that repositions the unattached head to search for a new forward binding site on actin^16,17^. This process repeats, resulting in multiple ATP-dependent steps on actin per diffusional encounter of a myosin-5a molecule^10,15,18^. Despite this consensus, it is controversial what size steps myosin-5a takes along actin, what role actin structure plays in the pattern of myosin-5a movement and what is the structural basis of gating of ADP release.

F-actin structure dictates the options for stepping by myosin-5a. It is a polar, helical polymer of globular actin subunits in which a single, left-handed, short-pitch helix is formed by successive subunits having an axial separation of 2.75 nm and a more variable left-handed rotation of ~-166°^19,20^. In side-view, this gives an appearance of two coaxial, right-handed helices of subunits, staggered by a half subunit, that cross over one another every ~36 nm (Figure 1B). For myosin-5a to walk straight, i.e., in a single azimuthal plane, along F-actin it must attach to a sequence of subunits of similar azimuth. These will generally be 13 subunits (35.75 nm) apart along the filament axis, because 13 × −166° is close to an integer number of turns (6 turns) of the short-pitch helix. When the trailing motor domain of a straight-walking molecule lets go, moves past the leading motor, and reattaches, the ‘stride’ that that motor takes is thus typically 26 subunits (71.50 nm), with each motor making 13-subunit steps ahead of its partner motor and the whole molecule making a forward movement of 13 subunits (35.75 nm). Myosin-5a can take strides of this length because of its long levers. The inherent polarity of the actin filament^21^ dictates the direction of movement of the myosin.

The structure of myosin-5a bound to actin by both motors while walking slowly in the presence of rate-limiting, micromolar ATP was captured by negative-stain electron microscopy (nsEM)^6^. The trailing head lever angle resembled the well-known ‘rigor’ complex formed by myosin heads on actin in the absence of ATP. By contrast the lead head lever emerged from the rear side of the leading motor domain and pointed backwards to meet the trailing head lever at their junction with the tail. The lead motor appeared to be in a near-primed position expected of a motor at the start of its working stroke, whereas the trail head converter was at the end of the working stroke^7^. However, this interpretation has not been universally adopted, in part because the measured rapid release of phosphate from the lead motor is associated with a motor transition to the unprimed state, and partly because of concerns that nsEM may have produced an artifactual structure. We have therefore used cryogenic electron microscopy (cryoEM) to reinvestigate the structure of the walking molecule. CryoEM avoids many possibilities for artifacts that are possible for nsEM because the sample is flash-frozen during activity without adsorption to a carbon film, fixation, staining or drying^22,23^, but it has not previously been successful for studying myosin-5a walking on actin.

Evidence suggests that myosin-5a must vary its stride length in order to walk straight. Recent high-resolution studies of F-actin^19,20,24,25^ find a subunit rotation of −166.5°, which implies a −4.5° (left-handed) movement around the actin filament per 13-subunit step. A run of 20 steps of 13 subunits, covering 715 nm, would thus entail a 90° rotation around the actin. Furthermore, nsEM of F-actin shows variable spacing of crossovers along each filament, which has been interpreted as arising from random variation in the rotation angle between successive subunits with a standard deviation of ~6°^26–28^. This cumulative angular disorder (CAD) means that the 13^th^ subunit will not always be best oriented to allow straight walking, so a variable stride would be necessary. Although CAD was demonstrated in early cryoEM studies of F-actin^29,30^, recent cryoEM studies have not commented on the presence of CAD in their specimens, leaving open to question whether CAD was merely an artifact of earlier preparation protocols. Although CAD in F-actin has been characterized, it has been unclear whether it has significance in the cell or is simply a biophysical curiosity. We have therefore analyzed recent cryoEM data of F-actin^19^ for evidence of CAD and find that it does indeed exist and has a major impact on myosin-5a stepping behavior.

It has been controversial whether myosin-5a takes uniform or variable steps as it walks along F-actin. nsEM indicated that the two heads of myosin-5a steps were commonly spaced 13 actin subunits apart but spacings of 11 and 15 actin subunits were also frequent^6,31^. In contrast, optical trapping or single molecule fluorescence assays of active molecules have shown a broad, continuous distribution of movements with a mean of ~37 nm or ~75 nm depending on the site and number of labels per molecule^10,32–37^. Recently, we used interferometric scattering (iSCAT) microscopy^38–40^ to study myosin-5a striding on actin^17^. A 20-nm gold nanoparticle coupled to the N-terminus of one motor (Figure 1B) enables precise localization (~1 nm) and high temporal resolution, in the order of milliseconds. The initial analysis of stride lengths again showed a single broad peak at 74 nm, consistent with previous studies. All these assays of active molecules have allowed a conclusion that all steps are 13 actin subunits, with a Gaussian broadening resulting from experimental and measurement uncertainties^13–15,32–34^. However, it was puzzling why the iSCAT peak was so broad given the method’s inherently high spatial resolution. We have therefore explored alternative methods of iSCAT data analysis and discovered that the breadth can be resolved into a family of discrete stride lengths.

The iSCAT study also detected a shift in position of the gold bead when the unlabeled head made its forward stride. This ‘AB transition’ is linked to the working stroke of the labelled motor that carries the unlabeled motor towards its next binding site (Figure 1B). It allows quantification of the dwell times of both heads of the molecule, and this showed that the kinetics of the labeled and unlabeled heads were indistinguishable. In our new analysis we exploit this phenomenon to yield a fuller understanding of myosin-5a stepping.

Here we study myosin-5a during its walk along actin filaments by combining cryoEM, nsEM and iSCAT microscopy. We show that myosin-5a is an inherently imprecise stepper. It exhibits multimodal step- and stride-length distributions that were not resolved by previous, lower resolution, single molecule stepping studies. We further show that single molecules of myosin-5a can walk straight, varying each step length to accommodate the variability in actin filament structure that is caused by CAD and thus do not exhibit step by step variations in their azimuthal position on the actin filament. The new EM data are consistent with our earlier proposal that a preferred angle between the two levers places the motors 36 nm apart along actin^17^, with an additional, thermally-driven elastic variation of this angle and in head shape allowing variable step size.

## Results

### iSCAT resolves varying stride lengths of myosin-5a

Molecules of myosin-5a construct labelled on the N-terminus of one motor with a 20-nm gold bead were tracked by iSCAT microscopy at 100 Hz as they walked along actin in the presence of 10 μM ATP. For each molecule, the x, y position of the bead was determined with sub-nanometer precision (0.9 nm localization precision)^17^ in each frame, allowing the stride and the AB transition to be identified (Figure 1C and E). The difference between mean positions of the bead in successive B-states yields the length of each stride taken by the labelled head (Figure 1B – D).

**Figure 2.**
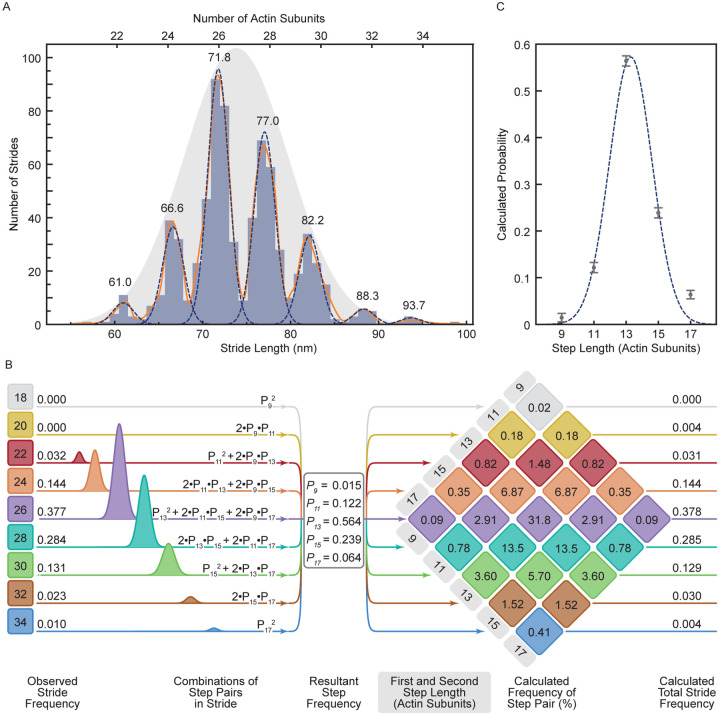
Analysis of strides taken by myosin-5a on F-actin. (A) Histogram of measured myosin-5 strides (N = 96 molecules, 725 strides). A Gaussian kernel density estimate, with locally adaptive bandwidth, is shown (solid orange line), as well as seven individual Gaussians (dashed lines). Mean stride length (nm) of each Gaussian is labelled above the fits, and calculated number of actin subunits traversed marked. The single Gaussian that expresses the overall mean and SD of the dataset is shown (grey shaded), rescaled to show fit to relative frequencies of the strides. (B) A schematic depicting the set of 9 equations used to calculate the probability of a given step length (P_x_, where x = 9, 11, 13, 15, 17 actin subunits) from the areas of the Gaussian fits to the stride peaks listed on the left side. Center panel lists the calculated step length probabilities. On the right is the multiplication diamond using the calculated probabilities of each step length to determine the percentage frequency of a stride resulting from each combination of step lengths. Combinations of step lengths that result in the same stride length are of the same color. The total predicted frequencies of each stride length are listed on the right for comparison with the observed frequencies listed on the left. (C) Step probabilities with fitted Gaussian.

The histogram of measured stride lengths is strikingly multimodal, with very little overlap between adjacent peaks (Figure 2A). This demonstrates unequivocally that myosin-5a takes strides of varying but quantized length and that iSCAT microscopy has the spatial precision to resolve these. The separation of adjacent peaks of 7 Gaussians fitted to the binned data is 5.45 ± 0.40 nm (mean ± SD), which corresponds to a separation of two actin subunits along the filament (5.5 nm), that is, it corresponds to neighboring subunits along one of the two long-pitch strands (Figure 1B). The major peak at 71.8 nm corresponds to a distance spanning 26 actin subunits (expected value, 71.5 nm). This is the stride length expected from two canonical 13-subunit steps. The other peaks therefore correspond to strides that land on subunits nearby to the 26^th^ along the same long-pitch actin strand. The family of peaks thus correspond to strides spanning 22, 24, 26, 28, 30, 32 and 34 actin subunits. Using the average of all the peaks, weighted by their normalized area, the spacing of subunits along F-actin is 2.758 ± 0.016 nm. Since the myosin motor binds stereo-specifically to actin^41^ the width of each peak likely arises from imprecision of measurement. This is expected to be independent of stride length and indeed an excellent fit to the data is obtained using a single width parameter (standard deviation (SD) = 1.195 nm) for all 7 peaks (Figure 2A).

Using iSCAT microscopy, it is therefore possible to describe any individual processive run by the precise numbers of actin subunits traversed in each of the strides. For example, the run shown in Figure 1C–E can be described as 30, 26, 28, 28, 24.

The data indicate that all molecules behaved similarly. Thus, comparison of the trajectories of the 96 molecules (Figure S2A) shows that all molecules take a range of stride lengths, rather than some being inherent short-striders and others, long-striders. Also, the distribution of stride lengths is independent of the number of strides taken in a processive run (Figure S2B). Furthermore, a *χ*^2^ analysis showed that the length of each stride was independent of the length of the preceding stride. These are all features to be expected if successive strides are independent events. The dataset of strides has an overall mean of 73.7 nm and SD of 5.66 nm. These overall values are close to those obtained in earlier studies using other methods that did not resolve the component peaks^32–34,37^. This indicates that the flexibility in stepping that we observe by iSCAT was also present in all those studies. This mean and SD can be represented by a Gaussian distribution and scaling its amplitude shows that it is a good fit to the relative amplitudes of the component peaks (Figure 2A). This indicates that the underlying cause of variable stride length has a Gaussian character. We investigate this further below.

It is important to note that the overall mean value of stride length falls between our observed peaks, and thus does not correspond to a stride length that is ever taken. This is because binding sites for motors on F-actin are not separated by this mean value. By using high resolution iSCAT microscopy, we have thus revealed that molecules of myosin-5a take strides of variable length during a single processive run.

### Myosin-5a step frequencies can be estimated from the stride frequencies

Quantitative analysis of the relative frequencies of each stride length (Figure 2B) reveals further insights into the stepping behavior of myosin-5a. Each stride length is the sum of a forward step by first the unlabeled motor and then the labelled motor. For example, a 24-subunit stride can result from 4 different combinations (1^st^ step, 2^nd^ step): (11, 13), (13, 11), (9,15), and (15,9) actin subunits (Figure 2B). Using the measured relative frequencies of the 7 stride lengths (areas under the fitted peaks; Figure 2A – B), we found the least squares best fit solution to the set of simultaneous equations containing the probabilities of the 5 underlying step lengths (motors spanning 9, 11, 13, 15, and 17-subunits) (Figure 2B, left panel). The fit was robust and excellent, with only small differences between the observed frequencies and those back-calculated from the fitted values of step probabilities (Figure 2B, right panel). The values of step probabilities versus step length are well fitted by a single Gaussian of mean 13.3 with SD 1.32 actin subunits (with associated fitting errors, 0.12 and 0.10 actin subunits, respectively) (Figure 2C). These properties are strong evidence that the walking process is the result of a random and independent selection of step lengths governed by an underlying Gaussian probability distribution.

The range of step sizes that is implicit in the multiple stride lengths shows that the canonical 13-subunit step is not as abundant as previously thought. Thus, only 56% of all steps are 13-subunits (Figure 2B, middle panel, and Table 1). From the calculated relative abundance of the various step lengths (Figure 2B), the average step length is 13.469 actin subunits (= 37.04 nm) (Table 1). Likewise, only 38% of the strides are 26-subunits, and this includes combinations of 11+15 subunit steps in addition to two 13-subunit steps. Only 32% of all strides are a combination of two consecutive 13-subunit steps (Figure 2B, right panel).

This analysis to determine step frequencies shows that myosin-5a is a more flexible stepper than has been widely recognized, and that the widely stated 36 nm step accounts for only about half of the steps taken.

### Structure of myosin-5a on actin using cryoEM

We used cryoEM for the first time to examine the structure of full-length myosin-5a during stepping on actin, to test the earlier conclusions from nsEM. In practice, it proved difficult to get these samples into the holes of the support film, but a small dataset has been obtained that delineates the structures present. In the presence of ATP, low calcium concentration and no cargo adaptor molecules, myosin-5a is mainly compactly folded^43–46^. These folded molecules are autoinhibited and bind only weakly to actin^46^. However a small fraction of molecules is found to be active and to move processively along actin^46^, so these should be present in samples flash frozen and examined by cryoEM. 55% of the myosin molecules observed were in the extended conformation and attached by both heads to the same actin filament (Figure S3). This abundance of myosin-5a molecules attached to actin is not surprising because we used a high ratio of myosin to actin (1 molecule per 2.5 actin subunits). Counts of myosin molecules per μm actin suggest that less than 10% were attached, which is consistent with the typical ~10-fold regulation of actin-activated ATPase activity in full-length myosin-5a preparations^44^. The relative scarcity of detached molecules may arise from their adsorption to the carbon film.

**Figure 3.**
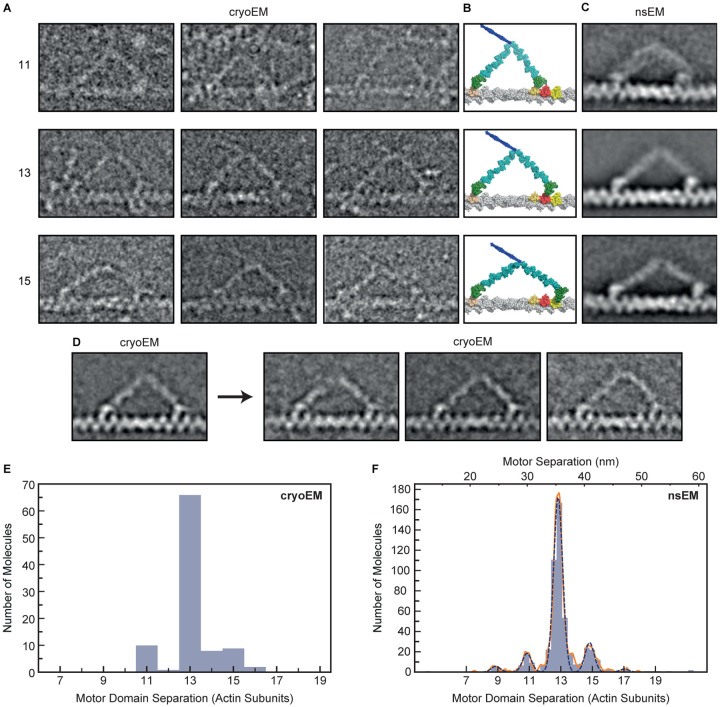
CryoEM and nsEM of myosin-5a walking on F-actin. (A) Gallery of single cryoEM images depicting myosin-5 molecules with step lengths of 11, 13 or 15 actin subunits. Contrast inverted (protein white). (B) Atomic models corresponding to (A), constructed according to Vale and Milligan^42^, assuming a 13/6 helix for F-actin. Actin subunits 11, 13 or 15 subunits away from the trailing motor domain are colored pink, red and yellow respectively. (C) Averaged nsEM images of myosin-5 molecules with step lengths of 11, 13 or 15 actin subunits. Note that for the step length of 11 subunits, the leading motor is in an unprimed conformation, unlike for 13 and 15 subunit steps. (D) Left panel: averaged cryoEM image of myosin-5 molecules with step length of 13 actin subunits. Right panels: result of classification into three subclasses based on features in the leading lever. Note that the right subclass has a convex leading lever shape, but the lever still emerges from the trailing side of the motor domain. (A-D) Actin barbed (+) end and myosin-5a leading head are on the right in all panels. All panels (A-D) are scaled to match the panels in (B), in which 13 actin subunits span a distance of 35.75 nm. (E) Measured separation between motor domains from cryoEM data. (F) Measured separation between motor domains from nsEM data. A Gaussian kernel density estimate, with locally adaptive bandwidth, is shown (solid orange line), as well as five individual Gaussians (dashed blue lines). The number of actin subunits is shown along the bottom, and the corresponding separation distance in nanometers along the top of the figure.

In raw cryoEM images, the motor domains, levers and the first, coiled-coil segment of the tail are frequently identifiable (Figure 3A). The two heads of doubly-attached molecules typically show asymmetry recalling the appearance seen in negative stain 6, with the two levers pointing in opposite directions. A global average of doubly-attached molecules with motor domains 13 actin subunits apart improves clarity (Figure 3D). Both motor domains are attached on the leading side of sub-domain 1 of actin (compare with atomic model, Figure 3B), with the N-terminal SH3-fold sub-domain extending as far forward as the axial position of sub-domain 1 of the next actin subunit, as in models of primed and unprimed heads on actin (Figure 3B)^47^. There is lower density between the motor and lever in the trail head than in the lead head, again in accord with the expectations from molecular models. The levers are visible throughout their length and unite at the head-tail junction without a gap, showing that the two polypeptide chains of the proximal coiled coil tail are not stretched apart by the stress within the doubly-attached molecule. All these features recall those seen using nsEM^6,31^.

In the trailing head the lever emerges from the leading side of the motor, near the SH3 domain, as expected for an unprimed head. The angle between the trailing lever and F-actin axis in the global average where the two motor domains are 13 subunits apart is 40°, as previously found by nsEM at rate-limiting, low ATP concentration (39–49°)^7^ in which the trailing heads were largely devoid of ADP. This indicates that release of ADP from the trailing head has little effect on the overall geometry of the doubly-attached myosin-5a, in contrast to the small lever swing found for single myosin-5a heads attached to actin when ADP is released^15,41^.

The leading heads show an abrupt angle between motor and lever and the lever emerges from the trailing side of the motor, indicating that the converter is in a near-primed position, despite the motor having released phosphate^7,48^ (Figure 3D). Classification of the leading lever region shows variation in shape, with some levers convexly curved away from the actin filament and emerging from the motor at ~120°, rather than straight and emerging from the motor at ~140° (Figure 3D, right panels). The curved levers still emerge from the trailing side of the motor domain, not the leading side. Thus in these cryoEM data, the leading motor domains are in a near-primed conformation, not unprimed.

The cryoEM data demonstrate that the observations made previously using nsEM^6,7^ are not artefacts of staining and drying. In particular the two motor domains of the doubly-attached molecule are in different structural states despite being in the same biochemical state. This may underlie their different rates of ADP release.

### Myosin-5a step size distributions from electron microscopy

Myosin-5a molecules with both heads attached on the actin filaments were identified and the separation between the two motor domains of the myosin molecule was measured by an unbiased method, whereby the positions of both motors were determined independently following alignment and classification. For this analysis it was not necessary to determine the polarity of the F-actin filament or to identify which of the two motors was the lead or trail head.

In the cryoEM dataset, motors are separated by 11, 13 and 15 actin subunits (Figure 3A). 77% of molecules have motors attached 13-subunits apart, 10% have motors 11-subunits apart and 13% are 15-subunits apart (Figure 3E). The mean separation is 13.115 actin subunits (= 35.87 nm) (Table 1).

A new dataset was also obtained using nsEM of the same myosin-5a construct as used for iSCAT and walking along phalloidin-stabilized F-actin under the same conditions as the iSCAT assay, including 10 μM ATP rather than the 1 μM ATP used in earlier studies^6,31^. This higher ATP concentration should produce a higher fraction of doubly-attached molecules that have ADP in the trailing motor domain (~50% at 10 μM ATP cf ~10% at 1 μM ATP). Analysis shows 9, 11, 13, 15 and 17-subunit separations (Figure 3C and F). Unlike in longer steps, the global image average of molecules stepping 11-subunits shows the leading head lever emerging from the leading side of the motor domain. This indicates that the smaller separation of motor domains allows the leading motor domain converter to move to the unprimed position, as found previously^31^ with concomitant distortion in the proximal lever. Like in the cryoEM data, 13-subunit steps are strongly favored (75%), with a mean step length of 13.044 actin subunits (35.87 nm) (Table 1). Thus phalloidin stabilization, which by increasing the rotation per actin subunit^49^ moves the 13^th^ subunit further from straight ahead, and brings the 15^th^ subunit closer to straight ahead, has little effect on the step-length probabilities.

The EM data complement the iSCAT data by showing the step lengths directly whereas the iSCAT data show strides, which each contain a pair of steps. Both EM methods show a reduced proportion of 15-subunit steps compared with iSCAT.

### How can a straight-walking myosin molecule be taking variable length strides?

**Figure 4.**
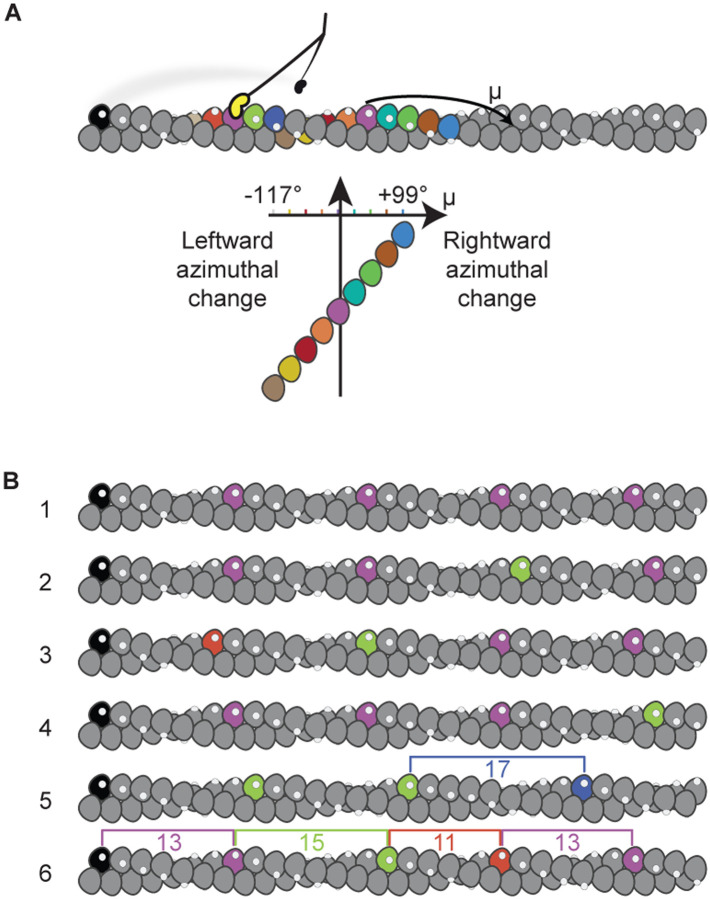
Implications of variable step length if F-actin has fixed or disordered helical parameters. (A) Schematic detailing how varying step and stride lengths would rotate myosin-5a trajectory around the actin filament given a fixed actin rotation per subunit, ϕ= −166.39°. Motor binding site on actin subunit depicted as white spot. A myosin-5a molecule is schematically shown at a stage where one motor (black) has detached from the black actin subunit and the attached motor (yellow) has undergone its power stroke. The detached motor is transiently dwelling off-axis, behind the plane of the figure and will bind to the right of the attached motor. Stride color scheme matches Figure 2. (B) F-actin with variable CAD. Filament 1 has fixed subunit rotation −166.39° and axial translation 2.75 nm. Filaments 2–6 were built incrementally from left to right by adding subunits with an addition to the rotation per subunit randomly drawn from a Gaussian distribution with mean 0° and standard deviation 5.28°. Actin subunits that lie closest to the same azimuth as the zeroth (black) subunit are highlighted with the step colors used in (A), to show expected myosin step lengths, indicated by brackets on filaments 5 and 6.

If F-actin was a perfectly regular helix, i.e., had a fixed rotation per subunit, our observation of randomly variable step length would imply that the myosin-5a molecule walks drunkenly along actin, moving left and right around the filament as well as along it (Figure 4A). However, in our iSCAT assay, myosin-5a walks straight along actin, staying perpendicular to the plane of the microscope coverslip to which the actin is bound (as described in Andrecka et al.; see also Methods Details). A possible solution to this paradox is that CAD in F-actin could force myosin-5a to vary its step length to continue walking straight. This would be equivalent to someone walking across a river using steppingstones that are variably spaced (Figure 4B). Thus, the relative frequencies of stride lengths would reflect the characteristics of CAD in the F-actin used in the iSCAT assay. We have therefore tested whether CAD in F-actin could be of sufficient magnitude to account for the observed striding behavior. In doing this we have enlarged upon two methods for assessing CAD from images of F-actin^26^, using recently published cryoEM images of F-actin^19^ (see Supplementary Information and Figures S4 to S6).

### Can CAD in F-actin account for the varying stride of myosin-5a?

**Figure 5.**
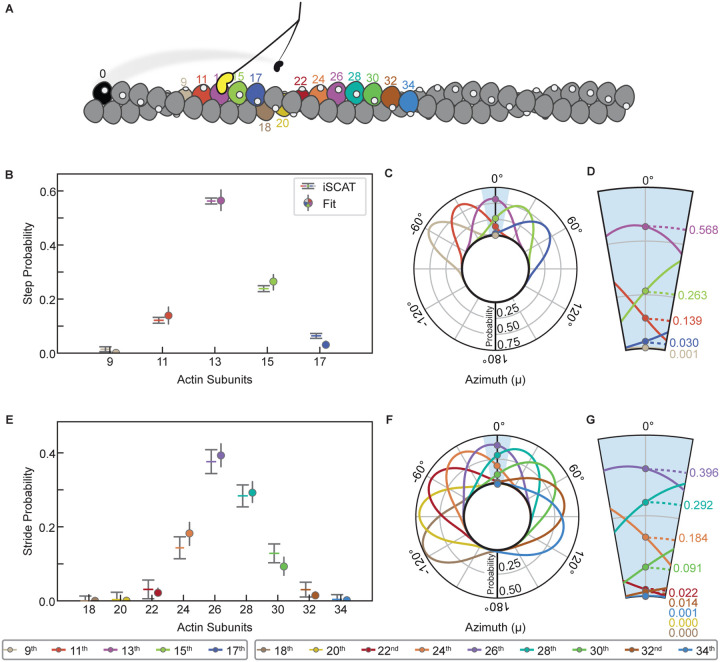
Comparison of F-actin cumulative angular disorder with myosin-5a step and stride length distributions. (A) Schematic numbering the actin subunits involved in steps and strides. Color scheme matches Figure 4. (B) and (E) Calculated probabilities of each step or stride length from iSCAT data, compared with probabilities from the angular disorder 0° azimuth fit, as shown in (C,D) and (F,G), respectively. (C) and (F) Radial plots, i.e., as if looking along the F-actin axis towards the barbed end, showing the azimuthal probability distributions of the n^th^ subunit away from the initial (i.e., the black) subunit along F-actin with the subunit rotation and CAD values (ϕ= −166.39 ± 5.28°) obtained from least squares fitting to the iSCAT data. Probability densities have been normalized such that the sum of probabilities at μ = 0° is 1. (D) and (G) Expanded ± 10° region showing probabilities of the n^th^ subunit lying at μ = 0°.

The implication of CAD for myosin-5a walking is as follows. The mean azimuth (μ_n_) of the n^th^ actin subunit, in degrees, relative to that of a starting subunit having an azimuth (μ_0_) of 0° is given by μ_*n*_ = *n*ϕ modulo 360, where ϕ is the mean rotation per subunit in the filament. The standard deviation (σ_n_) of that azimuth depends on the RMSD disorder value, *d*, and the number of subunits through $\sigma_{n}=d\sqrt{n}$ Thus, for any pair of values of ϕ and *d*, one can estimate the probabilities of the 9, 11, 13, 15 and 17-subunits being closest to an azimuth of 0° and thus the preferred target for straight walking (Figure 5A). It is important to appreciate that on the rare occasions when the 17^th^ subunit is optimally positioned, the 13^th^ will be at μ ≈ 55°, requiring a significant reach around the actin. We now apply these principles to test if CAD can account for the relative frequencies of each step and stride length.

If the variable step lengths in the iSCAT data were solely a response to CAD in actin, then our estimates of the relative frequencies of 9, 11, 13, 15 and 17-subunit steps should closely match the relative probabilities of those subunits being correctly positioned for straight walking due to CAD. We therefore used our five stepping frequencies to constrain least squares best fit estimates of ϕ and *d* in the set of 5 pairs of equations for μ_n_ and σ_n_ to test whether the resulting values agree with current estimates. A robust fit was found, yielding ϕ= −166.39° ± 0.11° and *d* = 5.28° ± 0.26°. Both these values are close to previous estimates, and there is an excellent match between the measured and fitted probabilities of the five step lengths (Figure 5B). Radial plots of the probability distribution illustrate the range of azimuth that each target actin subunit occupies, as viewed along the actin filament axis (Figure 5C). The enlarged segment around μ = 0° shows the azimuthal dependence of probability for straight walking (Figure 5D). For the 17^th^ subunit, the shallow slope of this dependence accounts for the relatively narrow error limits. We conclude that the presence of CAD in F-actin is sufficient to quantitatively account for the variable step lengths taken by myosin-5a.

If CAD is indeed the origin of variable step lengths in the iSCAT assay, then it should also account for the variable stride lengths. The ϕ and *d* parameters of the actin filament derived from stepping probabilities were therefore used to predict the relative probabilities of the 22^nd^ to 34^th^ subunit positions that are the target sites for the striding motor. These also give a very good match with the observed frequencies (Figure 5E). The radial plots of probability distribution (Figure 5F and G) are noticeably broader than those for the steps, demonstrating the progressive reduction of the correlation with the starting azimuth that is a feature of cumulative (liquid-like) disorder. This good fit to the striding data indicates that CAD in actin does indeed account fully for the spread and relative frequencies of strides observed in the iSCAT data.

Our analysis demonstrates that typical amounts of CAD in F-actin are sufficient to dictate a widely varying stepping pattern for a myosin-5a molecule walking straight.

### Step lengths found by EM indicate myosin-5a walks left-handed around free F-actin

The EM data give a complementary view of myosin-5a stepping to that of iSCAT, in that the myosin is walking along F-actin that is free in solution, rather than apposed to a surface, at the point of EM grid preparation. For the cryoEM dataset, longer steps are rarer than in iSCAT, indicating that myosin-5a prefers to take 13-subunit steps even when CAD means that it must move left-handed around the F-actin axis to do so. The average step length (13.115 actin subunits) is therefore shorter than the average iSCAT step length (13.469 subunits). As a result, using the value of −166.39° for mean actin rotation per subunit, derived above from the iSCAT data, yields an average azimuthal movement that is left-handed of 2.42° per step. This value implies that on average, myosin-5a would take 149 steps to complete a left-handed rotation around the filament, while moving 5.4 μm along it. The nsEM dataset is very similar, yielding an average step length of 13.044 subunits and corresponding values of 2.34°, 154 steps and 5.5 μm.

### Does step length influence myosin-5a ATPase kinetics?

**Figure 6.**
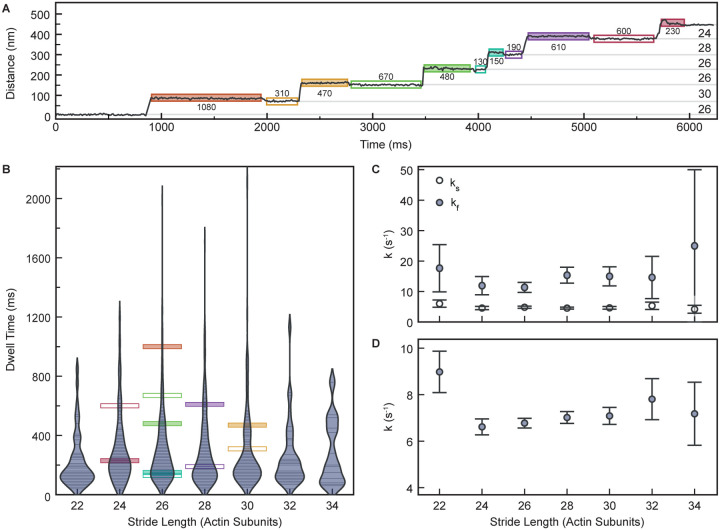
Analysis of impact of stride length on dwell times of A and B states. (A) Distance vs. Time trace of a typical gold-labelled myosin-5a motor domain demonstrating variability in dwell times of labelled motor domain. Dwell times (ms) marked below A states (filled boxes) and above B states (empty boxes). (B) Measured dwell time of A states after stride and B states before stride as a function of stride length. Each observed dwell is shown as a horizontal line. Violin plot envelopes are kernel density estimates of the distributions with bandwidth of 150 ms. (C) Fitted variable rate constants, *k*_*s*_ and *k*_*f*_, as a function of stride length. (D) Shared fit variable rate constant, *k*, as a function of stride length. Error bars in (C) and (D) denote the standard error of the fit parameters obtained from the observed Fisher information matrix.

When myosin-5a takes a long step, the levers necessarily lie at a smaller angle to the actin filament than when it takes a short step, as is indeed seen by EM (Figure 3). Since release of ADP from the head moves the lever to a smaller angle to actin^15,50^ it might be expected that when the motors are separated by more actin subunits, the rate of ADP release from the trail head would be accelerated, resulting in a shorter dwell time, than would be observed with shorter separations. Conversely, short steps suggest a larger lever angle, slower ADP release and longer dwell time. Because iSCAT resolves varying stride lengths, we have been able to test this idea. There is the complication that a stride may comprise a short step plus a long step in either order, but the shortest strides will comprise only short steps or longest strides only long steps. The two dwell times that refer to a given stride are those of the B state that precedes it and the A state that follows it (see Figure 1E and 6A). Therefore, we have analyzed these dwell times for each stride length.

We found that there was no significant difference between the dwell times for the A states compared to those of the B states, regardless of stride length, in agreement with our earlier study^17^, and thus confirming that the gold bead did not affect stepping kinetics. Violin plots of combined A-state and B-state data were similar across all stride lengths, with the most common stride lengths showing the greatest range of values, as expected (Figure 6B). Consequently, there was meagre evidence of dependence of rate constant on stride length (Figure 6C and D, and Figure S7), with rate constants for the two fitting models of *k*_*s*_ ≈ 4.5 s^−1^ and *k*_*f*_ ≈ 12 s^−1^ (Figure 6C and S7A) or *k* ≈ 7s^−1^ (Figure 6D and S7B) and no significant difference between the models as a fit to the data. We conclude that the walking rate, and thus ATPase kinetics, of myosin-5a is not greatly dependent on step length.

## Discussion

Using iSCAT microscopy we have succeeded in resolving a family of stride lengths taken along F-actin by myosin-5a that has one head labelled with a gold nanoparticle. The overall mean and standard deviation of the dataset are similar to those of earlier studies that did not resolve the component peaks, indicating that these variable strides are a constant feature of myosin-5a stepping.

Why were these variable stride lengths not resolved in previous studies? One seemingly trivial reason is that because the strides differ in length by one actin subunit distance (5.5 nm) it is necessary to aggregate the data into small enough bins (≤1.8 nm) to resolve that spacing, yet this has generally not been done. A second reason is that the method used to determine the start and end positions of each stride can add sufficient error that the peaks overlap. Thus, for the iSCAT data, a switch from measuring lengths from distance-time traces^17^ to measuring them from the x, y coordinates proved critical to resolving the strides. A further challenge arises where the center of mass of the molecule is being monitored, such as if both heads are labelled or in optical trap studies. Only if successive motor separations along actin are all the same is the movement of the molecule equal to the motor separation. If they differ, as we have shown they do, then in making the transition between them, the center of mass moves by the average of the two separations. For example, if a motor separation of 13-subunits is followed by one of 15-subunits, the center of mass moves 14-subunits. This means that the movement of the center of mass is not directly reporting the sizes of the steps taken by the motors. It also means that it is more demanding to resolve the subpeaks because they are only 2.75 nm apart.

### Misconception of the myosin-5a average step length explained

In contrast to common statements that myosin-5a walks by taking 36 nm steps along actin, we find that only about half the steps span the canonical 13-subunits (35.75 nm). Almost a quarter span 15-subunits with progressively smaller proportions spanning 11, 17 and 9-subunits. Because of this diversity of step lengths, neither the average step nor average stride length corresponds to an actual movement, as these average values fall between the separation of actin binding sites. These results from the iSCAT assay pertain also to previous assays that used F-actin attached to a coverslip or suspended between a pair of beads in the optical trap. This is because in each case only a limited azimuth of F-actin is available for the myosin to walk along. The presence of a family of unresolved steps or strides in the previous data explains why the breadth of the observed distribution was broader than would have been expected from the resolution of the methods used.

### Cumulative angular disorder of actin filaments accounts for myosin-5a step and stride frequency

We show that CAD exists within specimens of F-actin prepared by modern methods. Remarkably, CAD is of sufficient magnitude to account for the relative frequencies of the step and stride lengths found in the iSCAT data for myosin walking along a single azimuth of the actin filament. This nicely resolves the paradox of how myosin-5a could take steps of varying length while still walking straight on the helical F-actin. Recent high resolution cryoEM structures of F-actin^19,20^ omit this feature of F-actin structure, instead emphasizing a precise (average) value for subunit rotation (close to −166.5° per subunit). However to obtain high resolution, these studies restrict the reconstructed volume to a few subunits which reduces the impact of disorder, and when a longer segment of filament is used, resolution reduces^19^, as is expected from CAD.

Our study has revealed the biological significance of CAD in influencing the behavior of myosin motors. Although CAD has been well-characterized for F-actin purified from skeletal muscle^26,27^, there are no data on CAD in cytoskeletal actin isoforms^51^, and also no data for the influence of cytoskeletal tropomyosin or for CAD in actin filaments bundled by cross-linking proteins such as fascin or fimbrin. Although for some of these complexes there are data on filament flexibility, there is no established causal linkage between the magnitude of CAD and flexibility. Our study therefore indicates a need for further research to characterize CAD in the cellular environment and to understand the roles of CAD in determining cellular behavior. Understanding the temperature dependence of CAD magnitude and dynamics would also be beneficial for understanding the importance of CAD in F-actin under physiological conditions for warm- and cold-blooded animals.

### Myosin-5a walks left-handed around F-actin

Previous studies have directly shown that myosin-5a molecules can walk left-handed around a suspended actin filament that has been stabilized by phalloidin^52–54^. Phalloidin increases actin subunit rotation to −167°^49^. This moves the 13^th^ subunit to an average azimuth 11° left of straight ahead and correspondingly the 15^th^ subunit moves to be only 15° to the right. Thus, walking straight on such filaments requires a high proportion of 15 subunit steps. For myosin taking mainly 13 subunit steps, as we observe by both cryoEM and nsEM, the molecule would twirl left-handed around the filament more strongly than for phalloidin-free F-actin. This may account for the shorter pitch of twirling (≈2–3 μm)^52–54^ compared to our prediction of 5.5 μm. This can be compared to an average run length of ≈1.3 μm^35,55,56^, from previous studies. The earlier conclusion that left-handed twirling along phalloidin-stabilized F-actin implied that myosin took 11 and 13 subunit steps^52^ is incorrect, because it assumed −166.154° rotation per subunit for F-actin (i.e. 13/6 helical symmetry) rather than −167°.

### Structure of the walking myosin-5a molecule

It is perhaps surprising that this is the first report of a cryoEM study of myosin-5a walking on actin, but in practice we have found it difficult to get the walking molecules into the holes of the EM support film. Nevertheless, our small dataset clearly shows that the converter of the leading motor domain is held in a stressed, near-primed position through tethering to the trail head. Thus, the flash-frozen, unstained walking molecule appears similar to our previous studies using nsEM at very low ATP concentrations^6,31^, and also to the nsEM data presented here that were obtained at ten times higher ATP concentration than previously.

The ‘Telemark skier’ analogy used to describe the appearance of doubly-attached myosin-5a^6^ has since been interpreted by some to mean that both motor domains are in an unprimed conformation, with the lead lever bent back to create the knee of the skier^33^. The EM data presented here confirms that this is not the case. The leading lever is not bent. Instead, the knee of the leading leg of the skier is actually the lever-motor junction, with the lower leg being formed from the motor domain together with the actin subunit it is attached to and appearing angled backwards because the motor is attached to the leading side of the prominent subdomain 1 of actin. In contrast, in the trailing head, actin subdomain 1, the motor domain and the lever are all roughly colinear, creating the different shape resembling the trailing leg of the skier. As the powerstroke of myosin-5a is similar in distance to the canonical step length of 35.75 nm^57^, a primed myosin head could easily bind to create the next leading head, with the subsequent rapid release of phosphate^48^ generating stress within the doubly-attached molecule as the head is unable to adopt the preferred unprimed (post-powerstroke) structure of actomyosin.ADP.

Our nsEM, performed at the same ATP concentration as the iSCAT assay shows that for 11-subunit motor separation this tethering is insufficient to prevent converter movement to the unprimed position, with concomitant distortion of the leading lever but little change in the trailing lever (Figure 3C). This may explain why we find that the stepping rate is not slower for short steps, but it is unknown whether this also reduces gating of lead motor ADP release.

### Mechanical properties of myosin and impact on ATPase kinetics

Our analysis indicates that myosin-5a has to take variable length steps in the iSCAT assay because the sample geometry restricts azimuthal variation, forcing the motor domain to attach to the subunit of the disordered actin filament that is closest to straight ahead. Thus, the mechanical properties of the myosin cannot be directly assessed, only that there is sufficient compliance within the molecule to allow it to vary its step length over the range observed. However, a similar range of step lengths, albeit that 13-subunit steps are more strongly favored, is found by both cryoEM and nsEM, in which the myosin is free to explore around the actin filament to locate binding sites. This similarity indicates that myosin-5a has a preferred lever-lever angle that places the motors ≈36 nm apart, as we previously proposed from iSCAT data^17^. The greater frequency of 13-subunit steps in the EM data, unaffected by phalloidin stabilisation of F-actin, indicates that when the myosin is free to explore around the actin filament to overcome the effects of CAD, there is a still a preference for binding with that motor separation, reinforcing the idea of a preferred geometry for the singly-attached molecule.

The symmetrical Gaussian spread of the frequencies of shorter and longer steps further suggests a thermally-driven fluctuation in motor separation that allows both longer and shorter steps. This could arise from bending within the levers and at the lever-motor junctions and from fluctuations in the lever-lever angle. The standard deviation of the distribution of step lengths leads to an estimate for the stiffness of the molecule along the actin filament axis, through the Equipartition Principle, as stiffness, *κ* = *k*_*B*_*T/σ*^2^. For our nsEM data, σ= 3.289 nm (Table 1) yielding *κ* = 0.374 pN/nm. If we suppose that the lever-lever angle is fixed, then since the two heads are mechanically in series, the stiffness of each head would be 0.748 pN/nm. A similar standard deviation (3.0 nm) was reported by Oke^58^ also using nsEM, indicating a similar stiffness.

Because we have been able to examine the dependence of stepping kinetics on stride length, we can use this estimate of myosin-5a stiffness to compare our data with earlier optical trap measurements of the impact of external force on kinetics of ADP release and ATP binding by single myosin-5a heads^15,59,60^. Our EM data indicate that a 13-subunit motor separation has least strain. A 15-subunit separation implies a forward displacement of the lever-lever junction of 2.75 nm, and thus an assisting force on the trailing head of 2.75 × 0.374 = 1.03 pN. For a 17-subunit separation the force would be 2.06 pN, which would be expected to produce a marked acceleration of ADP release and thus reduction in B-state dwell time. It remains to be understood why this acceleration was not detected, but we note that in the iSCAT assay the orientation of the head with respect to the actin filament axis is closely specified (and relevant to myosin walking in the cell), whereas in the optical trap the orientation of the head is not known.

## Conclusions

Our demonstration of variable step and stride lengths for myosin-5a provides a framework for understanding how the molecule manages to transport cargoes through the complex and crowded cytoskeletal matrix. The steps the molecule takes are constrained by the mechanical properties of the myosin and, as we have revealed, also by CAD in F-actin. Unlike in macroscopic transport systems, both motor and track are ‘soft’, containing elements of random disorder that create adaptability.

## Supplementary Material

1

## Figures and Tables

**Table 1.** Calculated probabilities of each step length from iSCAT, cryoEM, and nsEM imaging. The mean step length and standard deviation in step lengths are also tabulated.

Step Length (actin subunits (asu))	Step Probability		
	iSCAT	cryoEM	nsEM
9	0.014		0.025
11	0.122	0.106	0.081
13	0.564	0.770	0.754
15	0.239	0.125	0.127
17	0.064		0.013
	iSCAT	cryoEM	nsEM
Mean Step (asu)	13.469	13.115	13.044
SD (asu)	1.585	1.035	1.196
Mean Step (nm)	37.039	36.066	35.871
SD (nm)	4.357	2.846	3.289
