# Supplementary material for: Myosin-5 varies its steps along the irregular F-actin track
Source: bioRxiv. 2023 Jul 16:2023.07.16.549178. Preprint. [Version 1] doi: 10.1101/2023.07.16.549178 (PMC10370000; doi:10.1101/2023.07.16.549178)
Supplement: 1 [file NIHPP2023.07.16.549178V1-supplement-1.pdf]

bioRxiv preprint doi: https://doi.org/10.1101/2023.07.16.549178; this version posted July 16, 2023. The copyright holder for this preprint (which was not certified by peer review) is the author/funder, who has granted bioRxiv a license to display the preprint in perpetuity. It is made available under aCC-BY 4.0 International license.

Supplementary Information

Characterizing cumulative angular disorder in F-actin CAD has not been mentioned in recent high-resolution studies of F-actin structure[1–4], we therefore first tested whether it could also be detected in F-actin prepared and imaged using current cryoEM methods. We analyzed recent high resolution cryoEM micrographs[1] from the EMPIAR archive, developing two tests for CAD[5] that make use of the spacings between crossovers of the two long-pitch helices of actin subunits. To properly interpret the results, we needed also to understand the impact on these tests of observer error in picking the position of crossovers in F-actin images, as this had not previously been evaluated. We therefore also analyzed test data from model actin filaments that were created in silico to have specified magnitudes of CAD (Figure 4B) and position error from picking crossovers (see Methods Details). Since both these variables were assumed to have a Gaussian spread of values distributed randomly along the filament, these simulations were done using large numbers of model filaments and the results averaged, in order to reduce the impact of the random fluctuations between filaments (Figure S5 and S6).

Test 1: Consecutive crossover correlation The first test examines the relationship between successive crossover spacings by creating a scatter plot from pairs of adjacent crossover spacings (Figure S4B). For a perfect helix, measured without error, all spacings will be identical (Figure S4B i). Introducing position error in the measurement, which generates a crossover spacing error (see Methods Details) along a perfect helix, the data points cluster into an ellipse with major axis having slope -1, because an overlong crossover estimate tends to be followed by a short one (and vice versa) (Figure S4C ii). The result is that the standard deviation of the data projected onto an axis of slope -1 (SD_{-1}) is much greater than that projected onto the orthogonal axis of slope +1 (SD_{+1}). The ratio of the two SDs (SD_{-1}/ SD_{+1}) is 1.73 (Figure S4C ii, inset), and is essentially independent of the magnitude of error from picking (Figure S5B). In contrast, the randomness inherent in CAD scatters the points in all directions from the mean center (Figure S4B iii). The non-linear relationship between crossover spacing and subunit rotation angle results in a SD slightly higher on the axis of slope +1 than on the orthogonal axis of slope -1, yielding a SD ratio of 0.97 (Figure S4C iii, inset). This value is, again, invariant with CAD magnitude (Figure S5D). As expected, a combination of CAD and position error produces an intermediate SD ratio between the two limiting values (Figure S4C iv). This modelling thus shows that an SD ratio below 1.73 is an indicator of CAD, but does not directly yield a value for CAD because the magnitudes of CAD and position error interact to determine the net SD ratio (Figure S5B and D).
In the cryoEM images, the polarity of each filament could be assigned by eye and the locations of the series of crossovers were clear (Figure S4D). For n = 371 crossovers picked in n = 54 actin filaments, the mean spacing ($\bar{x}$) was 37.95 nm with an SD of 2.19 nm. The mean spacing implies a mean rotation per subunit ($\overline{\phi}$) of -166.96°, close to the value (-166.85°) reported for these images[6]. The scatter plot of successive crossover spacings yielded a SD ratio of 1.36, well below the value for position error (1.73), and this thus indicates a strong contribution from CAD (Figure S4E).

Test 2: Cumulative variance The second test for CAD derives from its cumulative nature. For a family of filaments, the set of measurements of the distance along a filament between crossovers separated by a given number of crossovers will have a mean and SD. For a perfect helix picked with zero error, the SD will always be zero (Figure S4C i). Modelling confirms that when position error is added, the SD of crossover distance does not depend on how many crossovers intervene, because the magnitude of SD derives only from the position errors at the start and end crossover locations in the image (Figure S4C ii). For a helix with CAD there is additional variability because the crossover spacings really are shorter and longer. The further one progresses along the filament, the broader the range of possible crossover positions will be and thus the SD of crossover position rises with crossover number (Figure S4C iii). More detailed analysis of large numbers of long model filaments and using a range of CAD magnitudes confirms the rise in SD and also shows that the variance of crossover distance rises linearly with crossover number (Figure S6A and B). The SD of crossover distance rises linearly with CAD over the range 0 – 10° (Figure S5F). Position error increases the SD through an addition to the variance value. It thus adds a constant offset to the variance values and has no effect on the slope (Figure S6C). Helical geometrical arguments relate crossover spacing to subunit rotation and this in turn allows a least squares estimation of both CAD and position error (see Methods Details).
Applying this test to the cryoEM data, the SD of crossover distance does indeed increase with the number of crossovers traversed, confirming that CAD is present in the F-actin (Figure S4F). The data points are noisy because of the random fluctuations expected within the relatively small dataset (n=54 filaments analyzed). The least squares fit yielded an estimate of 2.24° per subunit for CAD, with a crossover error SD of 1.30 nm, implying a position error of 0.92 nm (Figure S5F). Applying these estimates to the first test (above) predicts a SD ratio of 1.31, close to the value of 1.36 observed.
Therefore, we conclude that CAD is an overlooked feature of F-actin. The SD of crossover spacing in this cryoEM dataset (2.19 nm) is smaller than in an early study (5.4 nm[7]), indicating that CAD magnitude is sensitive to experimental conditions.

Contact for reagent and resource sharing

Further information and requests for reagents and resources should be directed to and will be fulfilled by the lead contact, Yasuharu Takagi (takagiy@nih.gov).

bioRxiv preprint doi: https://doi.org/10.1101/2023.07.16.549178; this version posted July 16, 2023. The copyright holder for this preprint (which was not certified by peer review) is the author/funder, who has granted bioRxiv a license to display the preprint in perpetuity. It is made available under aCC-BY 4.0 International license.

KEY RESOURCES TABLE

REAGENT or RESOURCE	SOURCE	IDENTIFIER
Biological samples		
G-actin	Sellers lab	N/A
Calmodulin	Sellers lab	N/A
BirA	Avidity, LLC	Bulk BirA - Bulk BirA: BirA biotin-protein ligase bulk reaction kit
Sf9 cells	Thermo Fisher Scientific/Gibco™	Cat#: 11496015
MAX Efficiency™ DH10Bac Competent Cells	Thermo Fisher Scientific/Gibco™	Cat#: 10361012
**Chemicals, peptides, and recombinant proteins**		
AviTag-Myosin-5a-HMM-eGFP-FLAG	This study/James R. Sellers lab	N/A
Full-length Myosin-5a	This study/James R. Sellers lab	N/A
20-nm gold particles with streptavidin	BBI, U.K.	Cat#: EM.STP20/1
PEG-PLL	SuSoS AG, Switzerland	Cat#: PLL(20)-g[3.5]-PEG(2)
$MgCl_2$	MilliporeSigma	Cat#: M8266
KCl	MilliporeSigma	Cat#: P3911
EGTA	MilliporeSigma	Cat#: E4378
MOPS	MilliporeSigma	Cat#: M3183
DTT	Calbiochem	Cat#: 04856126
PMSF	Calbiochem	Cat#: 52332
PBS	Thermo Fisher Scientific/Gibco™	Cat#: 10010023
Sodium azide	MilliporeSigma	Cat#: S8032
1% uranyl acetate	Electron Microscopy Services	Cat#: 541-09-3
ATP	MilliporeSigma	Cat#: A2383
BSA	MilliporeSigma	Cat#: A0281
Phalloidin	MilliporeSigma	Cat#: P2141
Sf9 cell media (Sf-900™ II SFM)	Thermo Fisher Scientific/Gibco™	Cat#: 10902088
**Critical commercial assays**		
BirA ligase kit	Avidity, LLC	Bulk BirA - Bulk BirA: BirA biotin-protein ligase bulk reaction kit
**Recombinant DNA**		
AviTag-Myosin-5a-HMM-eGFP-FLAG	This study/James R. Sellers lab	N/A
Full-length Myosin-5a	This study/James R. Sellers lab	N/A
**Software and algorithms**		
ImageJ	Schneider *et al.*[8]	https://imagej.nih.gov/ij/
Python	Python Software Foundation	https://www.python.org/
LabVIEW	National Instruments	https://www.ni.com/en-us/shop/labview.html
GraphPad Prism	GraphPad by Dotmatics	https://www.graphpad.com/

bioRxiv preprint doi: https://doi.org/10.1101/2023.07.16.549178; this version posted July 16, 2023. The copyright holder for this preprint (which was not certified by peer review) is the author/funder, who has granted bioRxiv a license to display the preprint in perpetuity. It is made available under aCC-BY 4.0 International license.

REAGENT or RESOURCE	SOURCE	IDENTIFIER
Spider		https://zenodo.org/record/1447157#.ZHdpw3bML-g
Matplotlib	Caswell *et al.*[9]	https://doi.org/10.5281/zenodo.592536
NumPy	Harris *et al.*[10]	https://doi.org/10.1038/s41586-020-2649-2
Pandas		https://doi.org/10.5281/zenodo.3509134
Seaborn	Waskom *et al.*[11]	https://doi.org/10.5281/zenodo.592845
SciPy	Gommers et al.[12]	https://doi.org/10.5281/zenodo.595738
Shapely	Gillies *et al.*[13]	https://doi.org/10.5281/zenodo.5597138
LMFit	Newville *et al.*[14]	https://doi.org/10.5281/zenodo.598352
MPMath	Johansson *et al.*[15]	https://doi.org/10.5281/zenodo.1476881
AdaptiveKDE	Shimazaki and Shinomoto[16]	http://dx.doi.org/10.1007/s10827-009-0180-4
Numdifftools	Brodtkorb and D'Errico[17]	https://pypi.org/project/numdifftools/
Statsmodels	Seabold and Pertkold[18]	https://www.statsmodels.org/stable/index.html
**Other**		
iSCAT microscope	This study/Philipp Kukura lab; Ortega Arroyo *et al.*[19]	https://www.nature.com/articles/nprot.2016.022
Electron microscope	JEOL	JEOL 1200EX
Cryo-electron microscope	FEI	FEI F20 FEG
Calibration grid	Thorlabs	R1L3S5P
Carbon film on copper grids for nsEM	Ted Pella	Cat#: 01840-F
Lacey Carbon Films on 400 Mesh Copper Grids	Agar Scientific	Cat#: AGS166-4

Experimental model and subject details

Plasmid DNA for recombinant protein was amplified in E. coli DH5a strain (Thermo Fisher Scientific) in LB medium at 37°C overnight. Plasmid DNA was transformed into DH10Bac E. coli cells (Thermo Fisher Scientific) and then used to make baculoviral stock per the manufacturer protocol with minor modifications[20]. Recombinant proteins were overexpressed using the Sf9/baculovirus expression system using the Sf900 II medium (Thermo Fisher Scientific). The cells were grown in a temperature-controlled incubator shaker at 27°C/90 RPM in 2.8 L Fernbach flasks without baffles for 48 - 60 hrs. Sf9 cells were collected into a 50 ml conical tube and flash frozen with liquid nitrogen. Cell pellets were stored in the -80 °C freezer for long-term storage until purification was performed.

Methods Details

Protein purification Rabbit skeletal muscle G-actin was prepared as described[21] and stored in liquid nitrogen until use. For cryoEM and nsEM, F-actin was prepared by a two-step procedure in which the divalent cation in the G-actin is first exchanged from Ca^{2+} to Mg^{2+} by addition of 0.1 volume of exchange buffer (3 mM $MgCl_2$, 11 mM EGTA, pH 7.0) in ice for 15 minutes, then polymerized by addition of 0.1 volume of polymerization buffer (300 mM KCl, 12 mM $MgCl_2$, 1mM EGTA, 120 mM MOPS, pH 7.0), yielding a solution composition of 25 mM KCl, 1.25 mM $MgCl_2$, 1.0 mM EGTA, 0.17 mM ATP, 10 mM MOPS, 0.17 mM Tris, pH 7.0.

bioRxiv preprint doi: https://doi.org/10.1101/2023.07.16.549178; this version posted July 16, 2023. The copyright holder for this preprint (which was not certified by peer review) is the author/funder, who has granted bioRxiv a license to display the preprint in perpetuity. It is made available under aCC-BY 4.0 International license.

For the iSCAT measurements and nsEM, a mouse myosin-5a HMM-like construct ((i.e. having the two heads together with the proximal coiled coil, like heavy meromyosin derived from muscle myosin-2; myosin-5a sequence amino acid sequence 1-1090) with an N-terminal AviTag biotinylation sequence[22,23] and C-terminal eGFP and FLAG sequence was expressed in the presence of calmodulin, using the Sf9/baculovirus expression system and purified as described previously[24]. The following is the amino acid sequence of the construct (total: 1352 amino acids):

MGLNDIFEAQKIEWHEMAASELYTKFARVWIPDPEEVWKSAELLKDYKPGDKVLLLHLEEG
KDLEYRLDPKTGELPHLRNPDILVGENDLTALSYLHEPAVLHNLRVRFIDSKLIYTYCGIVLV
AINPYEQLPIYGEDIINAYSGQNMGDMDPHIFAVAEEAYKQMARDERNQSIIVSGESGAGKTV
SAKYAMRYFATVSGSASEANVEEKVLASNPIMESIGNAKTTRNDNSSRFGKYIEIGFDKRYRII
GANMRTYLLEKSRVVFQAEEERNYHIFYQLCASAKLPEFKMLRLGNADSFHYTKQGGSPMIE
GVDDAKEMAHTRQACTLLGISESYQMGIFRILAGILHLGNVGFASRDSDSCTIPPKHEPLTIFC
DLMGVDYEEMCHWLCHRKLATATETYIKPISKLQATNARDALAKHIYAKLFNWIVDHVNQ
ALHSAVKQHSFIGVLDIYGFETFEINSFEQFCINYANEKLQQQFNMHVFKLEQEEYMKEQIPW
TLIDFYDNQPCINLIESKLGILDLLDEECKMPKGTDDTWAQKLYNTHLNKCALFEKPRMSNK
AFIIKHFADKVEYQCEGFLEKNKDTVFEEQIKVLKSSKFKMLPELFQDDEKAISPTSATSSGRT
PLTRVPVKPTKGRPGQTAKEHKKTVGHQFRNSLHLLMETLNATTPHYVRCIKPNDFKFPFTF
DEKRAVQQLRACGVLETIRISAAGFPSRWTYQEFFSRYRVLMKQKDVLGDRKQTCKNVLEK
LILDKDKYQFGKTKIFFRAGQVAYLEKLRADKLRAACIRIQKTIRGWLLRKRYLCMQRAAIT
VQRYVRGYQARCYAKFLRRTKAATTIQKYWRMYVVRRRYKIRRAATIVIQSYLRGYLTRNR
YRKILREYKAVIIQKRVRGWLARTHYKRTMKAIVYLQCCFRRMMAKRELKKLKIEARSVER
YKKLHIGMENKIMQLQRKVDEQNKDYKCLMEKLTNLEGVYNSETEKLRNDVERLQLSEEEA
KVATGRVLSLQEEIAKLRKDLEQTRSEKKSIEERADKYKQETDQLVSNLKEENTLLKQEKET
LNHRIVEQAKEMTETMERKLVEETKQLELDLNDERLRYQNLLNEFSRLEEVSKGEELFTGVV
PILVELDGDVNGHKFSVSGEGEGDATYGKLTLKFICTTGKLPVPWPTLVTTLTYGVQCFSRYP
DHMKQHDFFKSAMPEGYVQERTIFFKDDGNYKTRAEVKFEGDTLVNRIELKGIDFKEDGNIL
GHKLEYNYNSHNVYIMADKQKNGIKVNFKIRHNIEDGSVQLADHYQQNTPIGDGPVLLPDN
HYLSTQSALSKDPNEKRDHMVLLEFVTAAGITLGMDELYKDYKDDDDK

Color labelling of amino acid sequence: AviTag, M5a motor domain, M5a SH3 domain, M5a IQ 1 - 6 motifs, M5a coiled-coil region, eGFP, and FLAG sequence.

For cryoEM, the melanocyte-specific isoform of full-length mouse myosin-5a heavy chain[25] was expressed together with calmodulin in Sf9 cells, purified using an N-terminal FLAG tag, dialyzed against 100 mM KCl, 0.1 mM EGTA, 1 mM DTT, 0.1 mM PMSF, 10 mM MOPS, pH7.0 and stored drop-frozen in liquid nitrogen, similar to as described before[24]. It was incubated with 4 moles of purified calmodulin per mole myosin overnight on ice, prior to cryoEM data collection.

Single molecule motility assay using iSCAT microscopy Biotinylation of the myosin-5a HMM construct ($\sim 100 \mu l$ of $\sim 4 - 5$ µM) with BirA ligase (Avidity, Aurora, Colorado), was performed on ice for ~12 hours as described in detail by the manufacturer protocol (https://www.avidity.com/showpdf.asp?N=B9B6C28E-1976-4CFE-89DE-E37D1D8DB2CA) . After this reaction, dialysis against 1 L of buffer containing 50 mM KCl, 0.1 mM EGTA, 1 mM DTT, 10 mM MOPS, pH 7.3, was performed for ~30 minutes (2x; total ~1 hr), to remove excess biotin. Biotinylated myosin-5a HMM was then used immediately for the iSCAT assay, or aliquoted and frozen in liquid nitrogen and stored in either liquid nitrogen storage or the -80 °C freezer for future use.

Newly biotinylated or stored myosin-5a HMM sample (750 nM per molecule) was diluted 10x in motility buffer (MB; 40 mM KCl, 5 mM $MgCl_2$, 0.1 mM EGTA, 5 mM DTT, 20 mM MOPS, pH 7.3) supplemented with 0.1 mg/ml BSA and 5 µM calmodulin. This was kept as a stock myosin solution (75 nM), on ice for the day of the experiment.

To form the stock myosin-5a HMM/gold nanoparticle complex, 0.77 nM streptavidin-conjugated 20 nm gold nanoparticles in tris-buffered saline (pH 8.2) containing 10 mg/ml BSA and 15 mM NaN_3 (BBI, UK) was incubated with 0.25 nM biotinylated myosin-5a HMM in MB. Thus, the gold:myosin ratio was ~1:0.3, similar to previous published methods[26,27] ensuring that on average, one or no myosin-5a molecule attached per gold nanoparticle. Myosin-5a HMM/gold nanoparticle complex was incubated on ice for approximately 15 min, immediately before preparing the final assay mix. New complexes were made prior to preparing a new flow cell for the assay.

20 µM F-actin stock solution was prepared in polymerization buffer (50 mM KCl, 1 mM $MgCl_2$, 1 mM EGTA, 10 mM imidazole (pH 7.3), 1.7 mM DTT , 3 mM ATP) and diluted with MB to 0.2 - 1 µM prior to use.

The flow cell (volume ~20 µl) was prepared as described[28,29], first rinsing with 1 mg/ml poly-(ethylene glycol)-poly-L-lysine (PEG-PLL) branch copolymer (SuSoS AG, Switzerland) in PBS (1 mM KH_2PO_4, 155 mM NaCl, 3 mM Na_2HPO_4-$7H_2O$, pH 7.4; Gibco) and incubated for 30 min. The flow cell was washed twice with MB (100 µl per wash) before adding the F-actin.

bioRxiv preprint doi: https://doi.org/10.1101/2023.07.16.549178; this version posted July 16, 2023. The copyright holder for this preprint (which was not certified by peer review) is the author/funder, who has granted bioRxiv a license to display the preprint in perpetuity. It is made available under aCC-BY 4.0 International license.

After F-actin was pipetted into the flow cell, attachment of the F-actin to the surface of the coverslip was monitored *via* the iSCAT microscope, as it can monitor label free proteins[30]. The chamber was then washed extensively with MB (500 µl) and the surface was blocked additionally with 1 mg/ml BSA in MB (100 µl). Prior to making the final assay mix, 0.1 M ATP (pH 7.0) was diluted 100x in MB to 1 mM ATP. This was then further diluted 100x to 10 µM ATP in the final assay mix.

The final assay composition in which stepping of myosin-5a HMM with a gold nanoparticle attached was observed, was as follows: 77 pM 20 nm gold nanoparticle attached with 25 pM myosin-5a HMM (*i.e.*, 10x dilution of previously mentioned stock), 10 µM ATP, 5 µM calmodulin, 40 mM KCl, 5 mM $MgCl_2$, 0.1 mM EGTA, 5 mM DTT, 20 mM MOPS (pH 7.3). Data collection was performed at room temperature (19 – 22 °C) and flow cells were not used for more than 30 minutes.

Description of iSCAT microscopy and data collection Interferometric scattering microscopy was performed as described previously[31,32]. In iSCAT microscopy, a 445 nm diode laser beam is scanned across the sample using a pair of orthogonal acousto-optic deflectors (Gooch & Housego) mapped into the back focal plane of the objective (PlanApo N 60x, 1.42 NA, Olympus) with a 4f telescope, giving an approximately 30 x 30 µm illumination. The illumination and detection paths are separated using a quarter-wave plate beneath the objective and polarizing beam splitter after the 4f telescope. The detected signal is imaged onto a CMOS camera (MV-D1024-160-CL-8, Photonfocus) at approximately 333× magnification (31.8 nm/pixel). The true magnification was determined using a calibrated resolution grid (Thorlabs) (Figure S1D).

Because of the potential for creating mirror inversions of the iSCAT image, both in the iSCAT microscope and in the analysis software, we confirmed that a clockwise, circular translation of the sample (as viewed from above the sample, *i.e.,* the coverslip on the far side of the actin filaments) resulted in a clockwise rotation of the iSCAT image and that this was unchanged by the software. By combining this information with the fact that myosin-5a always processes towards the barbed end of actin, we define left and right relative to an actin filament as if viewing the filament and motor from above, with the actin filament aligned along the y-axis and myosin walking up the y-axis.

nsEM sample preparation and data collection For nsEM, 20 µM F-actin was stabilized with 24 µM phalloidin. F-actin and the myosin-5a HMM construct used for iSCAT assays, but without the gold bead, were each diluted in 50 mM NaCl, 1 mM $MgCl_2$, 0.1 mM EGTA, 10 µM ATP, 10 mM MOPS, pH 7.0. Equal volumes of F-actin and myosin were then mixed to give final concentrations of 500 nM and 25 nM respectively. Samples were incubated for 30s, applied to UV-treated carbon film on copper EM grids and stained using 1% uranyl acetate. Micrographs were recorded on a JEOL 1200EX microscope using an AMT XR-60 CCD camera at 0.31 nm/pixel, calibrated using catalase crystals.

CryoEM sample preparation and data collection Full-length mouse myosin-5a in 100 mM KCl, 0.1 mM EGTA, 1 mM DTT, 10 mM MOPS, pH7.0 was incubated with 4 moles calmodulin per mole myosin overnight in ice. ATP was added from 7.8 mM stock solution to give 0.2 mM ATP, then mixed with a small volume of 83 µM F-actin and applied within 10 s to a lacey carbon grid (glow discharged in amylamine), quickly blotted with Ca-free paper and flash frozen in ethane slush. Final conditions: 0.83 µM myosin-5a, 3.6 µM calmodulin, 2.1 µM F-actin, 0.20 mM ATP, 95 mM KCl, 48 µM $MgCl_2$, 0.12 mM EGTA, 10 mM MOPS, pH 7.0 at 22°C. Specimens were imaged in an FEI F20 FEG cryoEM at ∼4 µm underfocus and recorded as 2x2k CCD images at 0.589 nm/pixel, calibrated with catalase crystal.

Quantification and Statistical Analysis

iSCAT stride length analysis iSCAT images were collected at 200 Hz and averaged down by a factor of 2 to identify the AB transitions (Figure 1C). The imaging frame rate was limited to 100 Hz (although iSCAT detection does allow much faster rates) to reduce the effects of label-linker flexibility, which increase localization noise at higher speeds and reduce our ability to identify the AB transition. An estimate of the localization precision achieved under these experimental conditions was determined by the positional fluctuations ($\sigma \sim 0.91$ nm, SD)[26] of a surface-attached label recorded under the same conditions as the trajectory in Figure 1C. For actin-bound myosin-gold conjugates the localization noise increases ($\sigma = 1.6 \pm 0.3$ nm)[26], but remains on average ∼2 nm which allows detection of the AB transitions on the order of 5 nm.

The pixel values of the gold particle were fitted with a 2-dimensional Gaussian to determine its x, y position (Figure 1C and D and Figure S1B and C) at nanometer precision. Myosin-5a strides were measured as the distance between the centers of two successive B states. The start and end of each B state was identified using a step detection algorithm[33]. The mean x, y coordinates ($\overline{x}_i$, $\overline{y}_i$) of each B state and the Euclidian distance between these mean x, y coordinates calculated:

$$Stride = S\left(\overline{x_1},\, \overline{x_2},\, \overline{y_1}, \overline{y_2}\right) = \sqrt{\left(\overline{x_2} - \overline{x_1}\right)^2 + \left(\overline{y_2} - \overline{y_1}\right)^2}$$

The data can be visualized as either an xy-plot (Figure 1C) or a distance-time trace (Figure 1E). To plot a distance time trace, the x and y values of the first data point in the run, at time = 0, (x_0, y_0) were subtracted from each of the values in the run (x, y) followed by addition in quadrature:

bioRxiv preprint doi: https://doi.org/10.1101/2023.07.16.549178; this version posted July 16, 2023. The copyright holder for this preprint (which was not certified by peer review) is the author/funder, who has granted bioRxiv a license to display the preprint in perpetuity. It is made available under aCC-BY 4.0 International license.

$$Distance = \sqrt{(x-x_0)^2+(y-y_0)^2}$$

However, distance-time traces do not adequately represent behavior during a dwell period because there is systematic underestimation of the contribution of bead movements orthogonal to the line joining the first data point to the mean center of the dwell position.

Myosin-5a strides, illustrated as blue arrows on the schematic, (Figure 1B) and highlighted as blue transitions on the example data trace (Figure 1C and E) were clear from these trajectories, as were the AB transitions, illustrated as red arrows on the schematic (Figure 1B) and highlighted as red portions on the example data trace, where they show as a decrease in distance (Figure 1C and E), as previously published[26]. The spread of particle localizations was calculated as the standard deviation of the gold particle during its stationary state (B-state, in this case) (Figure 1C), the uncertainty of particle positions is reported as the standard error of the mean (Figure 1D). The error on stride distances (σ_{Stride}) (Figure 1D) was calculated by error propagation of the standard errors of the means:

$$\sigma_{Stride} = \sqrt{\left(SEM_{x_1}\frac{\partial S}{\partial \overline{x_1}}\right)^2+\left(SEM_{x_2}\frac{\partial S}{\partial \overline{x_2}}\right)^2+\left(SEM_{y_1}\frac{\partial S}{\partial \overline{y_1}}\right)^2+\left(SEM_{y_2}\frac{\partial S}{\partial \overline{y_2}}\right)^2}$$

$$= \sqrt{\frac{SEM_{x_1}(\overline{x_2}-\overline{x_1})^2+SEM_{x_2}(\overline{x_2}-\overline{x_1})^2+SEM_{y_1}(\overline{y_2}-\overline{y_1})^2+SEM_{y_2}(\overline{y_2}-\overline{y_1})^2}{(\overline{x_2}-\overline{x_1})^2+(\overline{y_2}-\overline{y_1})^2}}$$

Orientation of myosin-5a in the iSCAT assay To fully understand the geometric implications of the experimental results, one must consider the geometry of the myosin-5a molecule relative to the experimental imaging axis. It is possible to rule out the scenario where the myosin-5a molecule binds and remains parallel to the glass surface, along the side of actin filament, for the entirety of its processive run. Due to BSA surface blocking, possible myosin binding sites on the side of the actin filaments are far more inaccessible as compared to those on the top. Binding of myosin-5a on top of the filaments will therefore minimize surface interactions with the gold label. Additionally, through experimental comparison of myosin-5a which has been labelled either at the motor or at the end of the coiled-coil domain, a significant contrast difference is seen between the motor-bound and coiled-coil-bound gold particles with the actin filament in focus. This difference in contrast is consistent with an approximately 40 – 60 nm difference in height above the actin[26]. Should myosin commonly bind and process on the side of the actin, parallel to the glass surface, no such contrast difference would have been observed.

Also, the off-axis unbound state reported in Andrecka et al.- Figure 8 has a mostly circular probability distribution. If a myosin-5a molecule started off parallel to the coverslip and rotated around the actin azimuth during its processive run, this distribution would become elliptical over the course of the run with the long axis at right angles to the actin filament axis. No such change in the distribution shape was observed. Also, as the myosin rotated around the actin the mean distance between the center of the 20 nm bead and the coverslip would vary from 10 – 25 nm above the glass surface. We would expect this to be accompanied by a change in signal contrast, however contrast of the bead shows little change between successive bound states[26].

For these reasons, together with those discussed in Andrecka et al. (specifically, in the section "Revealing the unbound head motion in three dimensions"), we determine that the myosin-5a in these experiments mostly binds and processively moves on top of the actin filament, perpendicular to the glass surface, with a limited range of angles bounded by the actin filament attachment to PEG-PLL/glass surface.

Stride distribution analysis Myosin-5a stride lengths were plotted as a histogram (Figure 2A) binned using a bin-width optimization algorithm[34]. The binned data were then fit with a sum of seven Gaussian functions, each with the same fixed width and different means and areas, using a least-squares method. The fixed width was determined as the value (1.195 nm) that minimized the error on the total fitted area of the sum of seven Gaussian functions. The probabilities of step lengths were calculated by a least squares best fit solution to the set of simultaneous equations containing the probabilities of the underlying step lengths. The KDE plot was created using an adaptive bandwidth algorithm[16].

To test whether consecutive strides were independent events, all pairs of consecutive strides (629 pairs from 96 runs that totaled 725 strides) were entered into a 9x9 grid (stride lengths 20 to 36 actin subunits). The relative frequency of each stride length was used to calculate the expected number of each pair for a chi-square test of the null hypothesis that strides are independent. There was no pattern to the distribution across the grid of differences between observed and expected values. Using the 22 cells for which the expected numbers were greater than 5, $p>0.05$, so the null hypothesis was accepted.

Myosin-5a cryoEM analysis Among 1,137 myosin-5a molecules identified in 57 micrographs, we found frequent (55%) extended molecules bound to F-actin by both motor domains (Figure S3), and small proportions bound by one motor domain (9%) or bridging between two actin filaments (4%). Very few (2%) compact molecules were associated with actin, as expected from our earlier work[35]. Remaining molecules were not attached to actin and were either extended (11%) or compact (18%).

bioRxiv preprint doi: https://doi.org/10.1101/2023.07.16.549178; this version posted July 16, 2023. The copyright holder for this preprint (which was not certified by peer review) is the author/funder, who has granted bioRxiv a license to display the preprint in perpetuity. It is made available under aCC-BY 4.0 International license.

Image processing comprised the following steps carried out within the SPIDER software package[36]. Images were contrast inverted (protein light) and CTF corrected by phase flipping. Decorated and undecorated F-actin filaments were segmented, aligned, and classified on features within actin to generate classes of polar filaments as judged by substructural detail. Those possessing bound myosin molecules were then selected (493 molecules) into a separate data set. To measure the distance between leading and trailing head motors along the actin filament we classified the images based on features around the motor domain, thus putting leading and trailing motor domains into different classes. We then marked the position of the motor domain (by eye) in averaged images of those classes where the motor domain was clearly visible. The distance between paired motors could then be computed from the raw images. Identifying the position of the motor domain was helped because the position of the actin subunits to which they are bound was very clear in these image averages. For generating averaged images with motors with different separations, having identified F-actin polarity (above) it was then necessary to mirror invert those images of myosin-bound molecules so that when displayed with F-actin running horizontally, all the myosin molecules were above the filament. This was done by image classification based on myosin features. Then all actin-bound myosin molecules were brought into a common alignment in which the molecules were walking to the right. Histograms were created using GraphPad Prism.
For generating averaged images with motors with different separations, having identified F-actin polarity (above) it was necessary to mirror invert those images of myosin-bound molecules so that when displayed with F-actin running horizontally, all the myosin molecules were above the filament. This was done by image classification based on myosin features. Then all actin-bound myosin molecules were brought into a common alignment in which the molecules were walking to the right.

Myosin-5a nsEM analysis Data analysis was conducted primarily in SPIDER software[36]. Molecules with both motors bound to actin were picked as two coordinate pairs, corresponding to both motor domain positions (n=1073 molecules). Using the coordinates, images were rotated to bring the actin horizontal with both motor domains positioned above the actin. Visual inspection of the stack of images was used to split the data into two, one subset with molecules walking right, one subset with molecules walking left. Leading heads from each dataset were subjected to reference-free alignment with restricted rotation and shift to create an aligned stack in which motor domains were superimposed. The same was done for trailing heads in each dataset. Each of the four aligned stacks was classified and divided into 20 classes per stack (= total of 80 classes) using K-means classification using a mask focused on the motor domain. A position was marked in the clearest class averages created from each dataset, corresponding to the contact point between the motor domain and actin (55/80 classes = 69% selected for defining contact points). These coordinates, along with the rotations and shifts from previous steps, were used to compute the location of the contact point in the original images and the motor-motor distance was calculated (n=566 molecules). Datasets were subdivided into classes based on motor-motor distances and averaging of these subclasses was used to validate the assignment of directionality and motor-motor distance measurement. Motor-motor distances were plotted as a histogram with adaptive bandwidth KDE (Figure 3F) using the same method as the iSCAT analysis (Figure 2A).

Analysis of cryoEM data for the presence of CAD CryoEM images of frozen hydrated chicken skeletal ADP-F-actin, at a resolution of 0.103 nm/pixel, were downloaded from EMPIAR dataset 11128[1]. Subunit detail was enhanced by Fourier bandpass Gaussian filtering between 50 and 20 pixels using Fiji-ImageJ 1.53. This allowed filament polarity to be assigned from the polar appearance between the crossovers (Figure S4D). Starting at the pointed (-) end of an uncluttered segment of an actin filament, consecutive crossover positions in a filament were marked by eye, using the Multipoint utility of Fiji-ImageJ, to create a dataset from n = 54 filaments. The sets of x, y coordinates generated for filament segments were further analyzed using Microsoft Excel to determine each crossover spacing and standard deviations of spacings. Distances from the starting crossover position to each subsequent crossover position were also computed. However, clutter sometimes limited the length of filament segment that could be used, with the result that crossover distances to the subsequent crossovers 1 – 4 could be measured on all 54 segments, but distances to crossovers 5, 6, 7, and 8 could be measured on only 51, 39, 28, and 18 segments respectively.

Creating model actin filaments Model filaments were built by sequential addition of subunits starting at the pointed (-) end, according to the following rules. The 3D coordinates of the centers of the nth actin subunit in a filament aligned along the x axis are given by the following set of parametric equations:

$$\begin{aligned} x_n &= n \cdot \Delta x \\ y_n &= \frac{r_f}{2} \sin\left(\mu_0 + n \cdot \overline{\phi} + d_n\right) \\ z_n &= \frac{r_f}{2} \cos\left(\mu_0 + n \cdot \overline{\phi} + d_n\right) \end{aligned}$$

where r_f is the filament radius, $\overline{\phi}$ is the mean rotation per subunit, d_n is the variable rotation per subunit, and Δx is the rise per subunit. $\overline{\phi}$ was set to -166.39°, r_f to 6.5 nm, d_0 to 0°, and Δx to 2.75 nm. For visualization, the azimuth of the initial

bioRxiv preprint doi: https://doi.org/10.1101/2023.07.16.549178; this version posted July 16, 2023. The copyright holder for this preprint (which was not certified by peer review) is the author/funder, who has granted bioRxiv a license to display the preprint in perpetuity. It is made available under aCC-BY 4.0 International license.

subunit (μ_0) is set to 60°, changing this initial value equates to a rotation of the whole filament around the x axis. For each sequential subunit the value of d_i was a random selection from a Gaussian distribution of values having a mean of 0° and a standard deviation of d. Visualization of these filaments (Figure 1B, 4A, 4B, and 5A) were created in Adobe Illustrator using the calculated coordinates as guides for actin subunit centers. Myosin motor binding site coordinates were estimated, for purposes of visualization, in a similar manner centered at r_f = 6.6 nm and setting the initial azimuth value to 110°.

Analysis of crossover distances of model filaments A side view of each model filament was studied by only considering the x and y coordinates. The filaments were modelled as two coaxial, long-pitched sinusoids by taking alternate subunits along the helical axis. The coordinates of the intersections of these two sinusoids were calculated using the Shapely package for Python[13]. If position error is included in the model, randomly generated noise from a Gaussian distribution with mean 0 nm and standard deviation of the position error was added to the intersection coordinates. Crossover distances were calculated by taking the Euclidean distance between adjacent crossovers.

Adjacent crossover spacings were plotted as scatter graphs (Figure S4B). To fit a regression line through this scatter, we used orthogonal distance regression (ODR) a specific case of Deming regression in which the error of variances in x and y are equal[12,37]. This yielded a slope of -1.00 $\pm$ 0.016. Therefore, to obtain a measure of the ellipticity of the scatter plot we computed the standard deviation of the data projected onto an axis of slope -1 (SD_{-1}) and the orthogonal axis of slope +1 (SD_{+1}). To do this, the (x, y) coordinates of the data points were first rotated clockwise by an angle $\theta = 45°$ about their mean using the following transformation matrix:

$$\begin{pmatrix} \cos\theta & \sin\theta \\ -\sin\theta & \cos\theta \end{pmatrix}$$

and then SD_{-1} and SD_{+1}were calculated as the SD of the y and x coordinates respectively. The ratio of the two SD values was calculated as SD_{-1}/SD_{+1}. Variation in this ratio for given conditions was determined through 1000 iterations and plotted as a Gaussian with mean and standard deviation of the ratios in this iterative dataset (Figure S4B, inset). Additionally, variation in the ratio as a function of both CAD and picking error was determined from 25 error values, evenly spaced, from 0 – 5 nm and 50 CAD values from 0 – 10° (Figure S5). The value of any one of these three values (ratio, CAD, error) can be determined from the other two by interpolation of this dataset using a Clough-Tocher 2D interpolator function[12].

The SD of distance along the model filaments as a function of crossover number was determined by taking the standard deviations of the Euclidean distance between each crossover and the first crossover of a sample of filaments (Figure S4C and Figure S6A). The variance is calculated as the square of these values (Figure S6B and C). A least squares linear fit to the variance is obtained[12].

Determining CAD and position error in EM data The average SD in crossover spacing due to the presence of CAD was calculated. From inspection of a helical net projection of the actin helix (Figure S4A), it is apparent from similar triangles that for a given mean crossover spacing, $\overline{x}$, as measured in the EM data, $\overline{x}$ / -180 = Δx / (-180 - $\overline{\phi}$). Thus, the mean rotation per subunit, $\overline{\phi}$, can be calculated:

$$\overline{\phi} \; = \; \frac{-180°\,(\overline{x} - \Delta x)}{\overline{x}}$$

For a given RMSD variable rotation per subunit, d, the RMSD CAD at the nth subunit, σ_n, is given by:

$$\sigma_n \; = \; d\sqrt{n}$$

Dividing σ_n by the mean crossover spacing, expressed in number of subunits, a mean change in rotation per subunit, $\Delta\phi_{\pm}$ can be obtained:

$$\Delta\phi_{\pm} \; = \overline{\phi} \, \pm \, \frac{\sigma_n}{\overline{x}}$$

This can be converted into a change in crossover spacing by substitution of $\Delta\phi_{\pm}$ into the equation for ϕ above.

$$\overline{x}_{\pm} = \; \frac{-180° \cdot \Delta x}{-180 - \; \Delta\phi_{\pm}}$$

The mean of the resulting values, $\overline{x}_{+}$and $\overline{x}_{-}$, is taken as the contribution CAD makes to the crossover spacing SD value. The square of this value will give the contribution to the variance, Var_{CAD}.

$$\overline{x}_{\pm}{}^2 = \; \text{Var}_{\text{CAD}}$$

Var_{CAD} scales approximately linearly with CAD over the range 0 – 10° (Figure S6F), although deviates from linearity at higher CAD values.

bioRxiv preprint doi: https://doi.org/10.1101/2023.07.16.549178; this version posted July 16, 2023. The copyright holder for this preprint (which was not certified by peer review) is the author/funder, who has granted bioRxiv a license to display the preprint in perpetuity. It is made available under aCC-BY 4.0 International license.

The contribution of error from picking to the observed variance of crossover spacings in the EM data, Var_{EM}, can thus be calculated as:

$$\text{Var}_{\text{Pick}} = \text{Var}_{\text{EM}} - \text{Var}_{\text{CAD}}$$

With the square root of this value giving the SD of error from picking for a crossover spacing. Since two crossover picks are made to determine a crossover spacing, the variance of each pick will be half that associated with the spacing measurement, and hence the SD for picking a crossover is (error from picking)/$\sqrt{2}$.
Through least squares fitting[12], an optimal CAD value can be obtained from the measured mean crossover spacing in a data set that produces a line of best fit of form $(n\ \text{Var}_{\text{CAD}} + \text{Var}_{\text{Pick}})$ (Figure S6E-F).

Actin disorder analysis For an actin filament with exactly 13 subunits in 6 turns of the left-handed short-pitch helix, the rotation around that helix per subunit, ϕ, (-166.154° in this case) is such that $13\phi = 6(-360)$. The difference in azimuth around the actin filament axis between the zeroth and the 13th subunit, $\mu_{13} = 13\phi - 6(-360)$. If ϕ has a larger value, μ_{13} will be negative. For the 11th subunit, $\mu_{11} = 11\phi - 5(-360)$. Generalizing for $0° < \mu_n < 360°$,

$$\mu_n = n\phi \text{ modulo } 360$$

It is convenient to set the zeroth subunit to 0°, and $-180° < \mu_n < 180°$. Adjusting the equation,

$$\mu_n = (n\phi + 180) \text{ modulo } 360 - 180$$

A characteristic of cumulative angular disorder is that the magnitude of disorder of a subunit is independent of the subunits around it. Therefore, the variance ($\sigma_n{}^2$) in subunit azimuth relative to a given starting subunit increases linearly with the number of subunits traversed, and thus:

$$\sigma_n = d\sqrt{n}$$

Where d is the RMSD disorder value in degrees.
Modelling an actin filament as a cylinder, a Gaussian azimuthal probability distribution of the nth motor binding location will be 'wrapped' around the circular cross section of the cylinder. This imposes a periodic boundary condition upon the distribution. Consequently, estimated azimuthal probability distributions (Figure 5C and F) for each step length (n) were calculated as wrapped normal distributions, with mean μ_n, using[38]:

$$f(\mu;\ \mu_n, \sigma_n) = \frac{1}{\sigma_n\sqrt{2\pi}} \sum_{k=-\infty}^{\infty} e^{\frac{-(\mu\ -\ \mu_n\ +\ 2\pi k)^2}{2\sigma_n{}^2}}$$

The distributions were rescaled by a common factor such that their values at an angle of $\mu = 0°$ summed to 1 (Figure 5D and G). Optimal ϕ and d values were obtained through least-squares fitting of the values of probability of each subunit being at zero azimuth to the iSCAT values of relative frequencies of step and stride lengths.

iSCAT dwell-time analysis The dwell-time analysis takes account of the fact that the duration of each step along actin is determined by the rate constants of ADP release from the trail head and the subsequent ATP binding to it that triggers trail head detachment from actin. Fitting of the dwell time measurements therefore uses the formula for a two-step reaction[39], in which k_1 conventionally refers to the second order ATP binding step and k_2 to the first order ADP release step, though these events occur in the reverse order in an attached head. At the concentration of ATP used in the iSCAT assay (10 µM), the observed first order rate constants of both processes are expected to be about 10 s^{-1} as discussed previously[26]. We therefore analyzed the dwell times using two models (Figure S6), one setting them to be equal, the other (arbitrarily) setting k_s to be less than k_f. In the latter case we are not specifying which of the two enzymatic steps has the smaller rate constant. Dwell times for the A and B states were pooled into a single dataset distinguished by the stride length preceding or succeeding the A and B state respectively. Rate constants for each stride length were determined through maximum likelihood fitting by minimizing a single global negative log-likelihood function for all strides. The distribution used is a convolution of two exponentials[40] of the form:

$$P(t) = \int_0^t k_s k_f e^{-k_s u} e^{-k_f (t-u)} du$$

if the two rates are not equal this evaluates to:

$$P_{k_s < k_f}(t) = \frac{k_s k_f}{k_f - k_s}(e^{-k_s t} - e^{-k_f t})$$

and if the two rate constants are equal, $k_s = k_f = k$:

bioRxiv preprint doi: https://doi.org/10.1101/2023.07.16.549178; this version posted July 16, 2023. The copyright holder for this preprint (which was not certified by peer review) is the author/funder, who has granted bioRxiv a license to display the preprint in perpetuity. It is made available under aCC-BY 4.0 International license.

$$P_k(t) = tk^2 e^{-kt}$$

Supplementary Bibliography

1. M. J. Reynolds, C. Hachicho, A. G. Carl, R. Gong, and G. M. Alushin. Bending forces and nucleotide state jointly regulate f-actin structure. *Nature*, 611(7935):380–386, 2022. ISSN 1476-4687 (Electronic) 0028-0836 (Print) 0028-0836 (Linking). doi: 10.1038/s41586-022-05366-w.
2. W. Oosterheert, B. U. Klink, A. Belyy, S. Pospich, and S. Raunser. Structural basis of actin filament assembly and aging. *Nature*, 611(7935):374–379, 2022. ISSN 1476-4687 (Electronic) 0028-0836 (Print) 0028-0836 (Linking). doi: 10.1038/s41586-022-05241-8.
3. F. Merino, S. Pospich, J. Funk, T. Wagner, F. Kullmer, H. D. Arndt, P. Bieling, and S. Raunser. Structural transitions of f-actin upon atp hydrolysis at near-atomic resolution revealed by cryo-em. *Nat Struct Mol Biol*, 25(6):528–537, 2018. ISSN 1545-9985 (Electronic) 1545-9985 (Linking). doi: 10.1038/s41594-018-0074-0.
4. S. Z. Chou and T. D. Pollard. Mechanism of actin polymerization revealed by cryo-em structures of actin filaments with three different bound nucleotides. *Proc Natl Acad Sci U S A*, 116(10): 4265–4274, 2019. ISSN 1091-6490 (Electronic) 0027-8424 (Print) 0027-8424 (Linking). doi: 10.1073/pnas.1807028115.
5. E. H. Egelman and D. J. DeRosier. Image analysis shows that variations in actin crossover spacings are random, not compensatory. *Biophys J*, 63(5):1299–305, 1992. ISSN 0006-3495 (Print) 1542-0086 (Electronic) 0006-3495 (Linking). doi: 10.1016/S0006-3495(92)81716-2.
6. R. Gong, F. Jiang, Z. G. Moreland, M. J. Reynolds, S. E. de Los Reyes, P. Gurel, A. Shams, J. B. Heidings, M. R. Bowl, J. E. Bird, and G. M. Alushin. Structural basis for tunable control of actin dynamics by myosin-15 in mechanosensory stereocilia. *Sci Adv*, 8(29):eabl4733, 2022. ISSN 2375-2548 (Electronic) 2375-2548 (Linking). doi: 10.1126/sciadv.abl4733.
7. A. Orlova and E. H. Egelman. F-actin retains a memory of angular order. *Biophys J*, 78(4):2180–5, 2000. ISSN 0006-3495 (Print) 1542-0086 (Electronic) 0006-3495 (Linking). doi: 10.1016/S0006-3495(00)76765-8.
8. C. A. Schneider, W. S. Rasband, and K. W. Eliceiri. Nih image to imagej: 25 years of image analysis. *Nat Methods*, 9(7):671–5, 2012. ISSN 1548-7105 (Electronic) 1548-7091 (Print) 1548-7091 (Linking). doi: 10.1038/nmeth.2089.
9. T.A. Caswell, A. Lee, M. de Andrade, E.S. Droettboom, T. Hoffmann, J. Klymak, J. Hunter, E. Firing, D. Stansby, N. Varoquaux, J.H. Nielsen, B. Root, R. May, O. Gustafsson, P. Elson, J.K. Seppänen, J-J. Lee, D. Dale, hannah, D. McDougall, A. Straw, P. Hobson, K. Sunden, G. Lucas, C. Gohlke, A.F. Vincent, T.S. Yu, E. Ma, S. Silvester, and C. Moad. matplotlib/matplotlib: Rel: v3.7.1, 2023.
10. C. R. Harris, K. J. Millman, S. J. van der Walt, R. Gommers, P. Virtanen, D. Cournapeau, E. Wieser, J. Taylor, S. Berg, N. J. Smith, R. Kern, M. Picus, S. Hoyer, M. H. van Kerkwijk, M. Brett, A. Haldane, J. F. Del Rio, M. Wiebe, P. Peterson, P. Gerard-Marchant, K. Sheppard, T. Reddy, W. Weckesser, H. Abbasi, C. Gohlke, and T. E. Oliphant. Array programming with numpy. *Nature*, 585 (7825):357–362, 2020. ISSN 1476-4687 (Electronic) 0028-0836 (Print) 0028-0836 (Linking). doi: 10.1038/s41586-020-2649-2.
11. M. Waskom, M. Gelbart, O. Botvinnik, J. Ostblom, P. Hobson, S. Lukauskas, D.C. Gemperline, T. Augspurger, Y. Halchenko, J. Warmenhoven, J.B. Cole, E. ter Hoeven, J. de Ruiter, J. Vanderplas, S. Hoyer, C. Pye, A. Miles, C. Swain, K. Meyer, M. Martin, P. Bachant, S. Molin, E. Quintero, G. Kunter, S. Villalba, Brian, C. Fitzgerald, C. Evans, M.L. Williams, and D. O'Kane. mwaskom/seaborn: v0.12.2 (december 2022), 2022.
12. R. Gommers, P. Virtanen, E. Burovski, M. Haberland, W. Weckesser, T.E. Oliphant, T. Reddy, D. Cournapeau, alexbrc, A. Nelson, P. Peterson, J. Wilson, P. Roy, endolith, I. Polat, N. Mayorov, S. van der Walt, M. Brett, D. Laxalde, Larson E., J. Millman, A. Sakai, Lars, peterbell10, P. van Mulbregt, CJ Carey, eric jones, N. McKibben, Kai, and R. Kern. scipy/scipy: Scipy 1.10.1, 2023.
13. S. Gillies, C. van der Wel, J. Van den Bossche, M.W. Taves, J. Arnott, B. C. Ward, and & others. Shapely (2.0.1), 2023.
14. M. Newville, R. Otten, A. Nelson, T. Stensitzki, A. Ingargiola, D. Allan, A. Fox, F. Carter, Michał, R. Osborn, D. Pustakhod, Ineuhaus, S. Weigand, A. Aristov, Glenn, C. Deil, mgunyho, Mark, A.L.R. Hansen, G. Pasquevich, L. Foks, N. Zobrist, O. Frost, Stuermer, azelcer, A. Polloreno, A. Persaud, J.H. Nielsen, M. Pompili, and S. Caldwell. lmfit/lmfit-py: 1.2.1, 2023.
15. F. Johansson, V. Steinberg, S.B. Kirpichev, K.L. Kuhlman, A. Meurer, O. Čertík, C. Van Horsen, P. Masson, J.A. de Reyna, T. Hartmann, M. Pernici, M. Kagalenko, P. Peterson, Z. Jędrzejewski-Szmek, S. Krastanov, J. Warner, W. Weckesser, T. Buchert, N. Schlömer, J. Creus-Costa, G.-L. Ingold, C. Behan, and A. Brys. mpmath: a python library for arbitrary-precision floating-point arithmetic. 2017. doi: https://doi.org/10.5281/zenodo.1476881.
16. H. Shimazaki and S. Shinomoto. Kernel bandwidth optimization in spike rate estimation. *Journal of Computational Neuroscience*, 29(1-2):171–182, 2010. ISSN 0929-5313. doi: 10.1007/s10827-009-0180-4.
17. P.A. Brodtkorb and J. D'Errico. Numdifftools, 2022.
18. S. Seabold and J. Perktold. Statsmodels: Econometric and statistical modeling with python. In *Proceedings of the 9th Python in Science Conference*. doi: https://www.statsmodels.org/stable/index.html.
19. J. Ortega Arroyo, D. Cole, and P. Kukura. Interferometric scattering microscopy and its combination with single-molecule fluorescence imaging. *Nat Protoc*, 11(4):617–33, 2016. ISSN 1750-2799 (Electronic) 1750-2799 (Linking). doi: 10.1038/nprot.2016.022.
20. J. E. Bird, Y. Takagi, N. Billington, M. P. Strub, J. R. Sellers, and T. B. Friedman. Chaperone-enhanced purification of unconventional myosin 15, a molecular motor specialized for stereocilia protein trafficking. *Proc Natl Acad Sci U S A*, 111(34):12390–5, 2014. ISSN 1091-6490 (Electronic) 0027-8424 (Print) 0027-8424 (Linking). doi: 10.1073/pnas.1409459111.
21. J. A. Spudich and S. Watt. The regulation of rabbit skeletal muscle contraction. i. biochemical studies of the interaction of the tropomyosin-troponin complex with actin and the proteolytic fragments of myosin. *J Biol Chem*, 246(15):4866–71, 1971. ISSN 0021-9258 (Print) 0021-9258 (Linking).
22. D. Beckett, E. Kovaleva, and P. J. Schatz. A minimal peptide substrate in biotin holoenzyme synthetase-catalyzed biotinylation. *Protein Sci*, 8(4):921–9, 1999. ISSN 0961-8368 (Print) 1469-896X (Electronic) 0961-8368 (Linking). doi: 10.1110/ps.8.4.921.
23. T. Lin, M. J. Greenberg, J. R. Moore, and E. M. Ostap. A hearing loss-associated myo1c mutation (r156w) decreases the myosin duty ratio and force sensitivity. *Biochemistry*, 50(11):1831–8, 2011. ISSN 1520-4995 (Electronic) 0006-2960 (Print) 0006-2960 (Linking). doi: 10.1021/bi1016777.
24. F. Wang, L. Chen, O. Arcucci, E. V. Harvey, B. Bowers, Y. Xu, 3rd Hammer, J. A., and J. R. Sellers. Effect of adp and ionic strength on the kinetic and motile properties of recombinant mouse myosin v. *J Biol Chem*, 275(6):4329–35, 2000. ISSN 0021-9258 (Print) 0021-9258 (Linking). doi: 10.1074/jbc.275.6.4329.
25. X. Wu, T. Sakamoto, F. Zhang, J. R. Sellers, and 3rd Hammer, J. A. In vitro reconstitution of a transport complex containing rab27a, melanophilin and myosin va. *FEBS Lett*, 580(25):5863–8, 2006. ISSN 0014-5793 (Print) 0014-5793 (Linking). doi: 10.1016/j.febslet.2006.09.047.
26. J. Andrecka, J. Ortega Arroyo, Y. Takagi, G. de Wit, A. Fineberg, L. MacKinnon, G. Young, J. R. Sellers, and P. Kukura. Structural dynamics of myosin 5 during processive motion revealed by interferometric scattering microscopy. *Elife*, 4, 2015. ISSN 2050-084X (Electronic) 2050-084X (Print) 2050-084X (Linking). doi: 10.7554/eLife.05413.
27. J. Andrecka, Y. Takagi, K. J. Mickolajczyk, L. G. Lippert, J. R. Sellers, W. O. Hancock, Y. E. Goldman, and P. Kukura. Interferometric scattering microscopy for the study of molecular motors. *Methods Enzymol*, 581:517–539, 2016. ISSN 1557-7988 (Electronic) 0076-6879 (Print) 0076-6879 (Linking). doi: 10.1016/bs.mie.2016.08.016.
28. A. R. Dunn and J. A. Spudich. Single-molecule gold-nanoparticle tracking. *Cold Spring Harb Protoc*, 2011(12):1498–506, 2011. ISSN 1559-6095 (Electronic) 1940-3402 (Print) 1559-6095 (Linking). doi: 10.1101/pdb.prot066977.
29. A. R. Dunn and J. A. Spudich. Dynamics of the unbound head during myosin v processive translocation. *Nat Struct Mol Biol*, 14(3):246–8, 2007. ISSN 1545-9993 (Print) 1545-9985 (Linking). doi: 10.1038/nsmb1206.
30. J. Ortega Arroyo, J. Andrecka, K. M. Spillane, N. Billington, Y. Takagi, J. R. Sellers, and P. Kukura. Label-free, all-optical detection, imaging, and tracking of a single protein. *Nano Lett*, 14(4): 2065–70, 2014. ISSN 1530-6992 (Electronic) 1530-6984 (Print) 1530-6984 (Linking). doi: 10.1021/nl500234t.
31. J. Ortega-Arroyo and P. Kukura. Interferometric scattering microscopy (iscat): new frontiers in ultrafast and ultrasensitive optical microscopy. *Phys Chem Chem Phys*, 14(45):15625–36, 2012. ISSN 1463-9084 (Electronic) 1463-9076 (Linking). doi: 10.1039/c2cp41013c.
32. Y. Takagi, N. Hundt, and A. Fineberg. Single-molecule biophysical techniques to study actomyosin force transduction. *Adv Exp Med Biol*, 1239:85–126, 2020. ISSN 0065-2598 (Print) 0065-2598 (Linking). doi: 10.1007/978-3-030-38062-5_6.
33. B. Kalafut and K. Visscher. An objective, model-independent method for detection of non-uniform steps in noisy signals. *Computer Physics Communications*, 179(10):716–723, 2008. ISSN 0010-4655. doi: 10.1016/j.cpc.2008.06.008.
34. H. Shimazaki and S. Shinomoto. A method for selecting the bin size of a time histogram. *Neural Comput*, 19(6):1503–27, 2007. ISSN 0899-7667 (Print) 0899-7667 (Linking). doi: 10.1162/neco.2007.19.6.1503.
35. K. Thirumurugan, T. Sakamoto, 3rd Hammer, J. A., J. R. Sellers, and P. J. Knight. The cargo-binding domain regulates structure and activity of myosin 5. *Nature*, 442(7099):212–5, 2006. ISSN 1476-4687 (Electronic) 0028-0836 (Print) 0028-0836 (Linking). doi: 10.1038/nature04865.
36. J. Frank, M. Radermacher, P. Penczek, J. Zhu, Y. Li, M. Ladjadj, and A. Leith. Spider and web: processing and visualization of images in 3d electron microscopy and related fields. *J Struct Biol*, 116 (1):190–9, 1996. ISSN 1047-8477 (Print) 1047-8477 (Linking). doi: 10.1006/jsbi.1996.0030.
37. P. T. Boggs, C. H. Spiegelman, J. R. Donaldson, and R. B. Schnabel. A computational examination of orthogonal distance regression. *Journal of Econometrics*, 38(1-2):169–201, 1988. ISSN 0304-4076. doi: Doi10.1016/0304-4076(88)90032-2.
38. D. Collett and T. Lewis. Discriminating between the von mises and wrapped normal distributions. *Australian Journal of Statistics*, 23(1):73–79, 1981. doi: 10.1111/j.1467-842X.1981.tb00763.x.
39. J. N. Forkey, M. E. Quinlan, M. A. Shaw, J. E. Corrie, and Y. E. Goldman. Three-dimensional structural dynamics of myosin v by single-molecule fluorescence polarization. *Nature*, 422(6930): 399–404, 2003. ISSN 0028-0836 (Print) 0028-0836 (Linking). doi: 10.1038/nature01529.

bioRxiv preprint doi: https://doi.org/10.1101/2023.07.16.549178; this version posted July 16, 2023. The copyright holder for this preprint (which was not certified by peer review) is the author/funder, who has granted bioRxiv a license to display the preprint in perpetuity. It is made available under aCC-BY 4.0 International license.

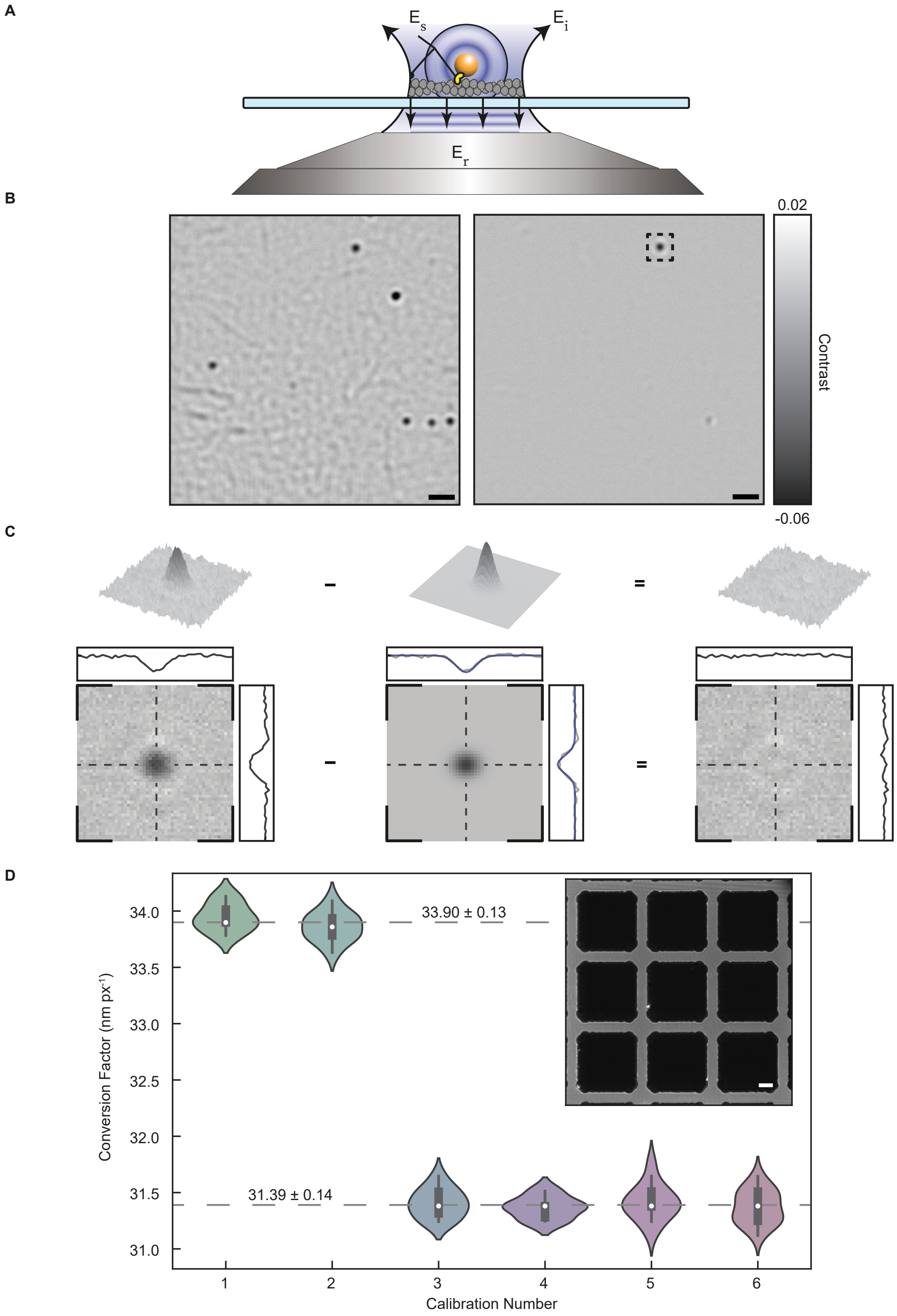

Figure S1. Essential principles of iSCAT microscopy. Related to Figure 1. (A) Schematic of iSCAT showing incident, reflected and scattered light (E_i, E_r, and E_s respectively). A coverslip (blue) supporting actin and myosin-5 labelled with a gold bead is imaged in an inverted microscope using an oil-immersion objective (grey). (B) (Left) Raw iSCAT image of unlabeled actin network with gold-labelled myosin-5a. (Right) Image with static background subtracted, which reveals only the gold nanoparticles that have moved since the static background image was recorded. Scale bar (black) = 1 µm (C) Image of the gold nanoparticle highlighted in (B) and the 2D Gaussian fit subtracted to show the fit residual. Data are shown as both an XY plus contrast axis plot and an image. (D) Calibration of iSCAT camera pixels. Calculated conversion factor from pixels to nanometers, as determined by use of a resolution grid (Inset; scale bar (white) = 2 µm). Microscope adjustments were carried out between calibrations 2 and 3. Violin plot envelopes are kernel density estimates of the distributions with bandwidth determined by Scott's rule of thumb. Grey dashed lines indicate two global means before and after microscope adjustment, together with standard error of mean.

40. A. Yildiz, J. N. Forkey, S. A. McKinney, T. Ha, Y. E. Goldman, and P. R. Selvin. Myosin v walks hand-over-hand: single fluorophore imaging with 1.5-nm localization. *Science*, 300(5628):2061–5, 2003. ISSN 1095-9203 (Electronic) 0036-8075 (Linking). doi: 10.1126/science.1084398.

bioRxiv preprint doi: https://doi.org/10.1101/2023.07.16.549178; this version posted July 16, 2023. The copyright holder for this preprint (which was not certified by peer review) is the author/funder, who has granted bioRxiv a license to display the preprint in perpetuity. It is made available under aCC-BY 4.0 International license.

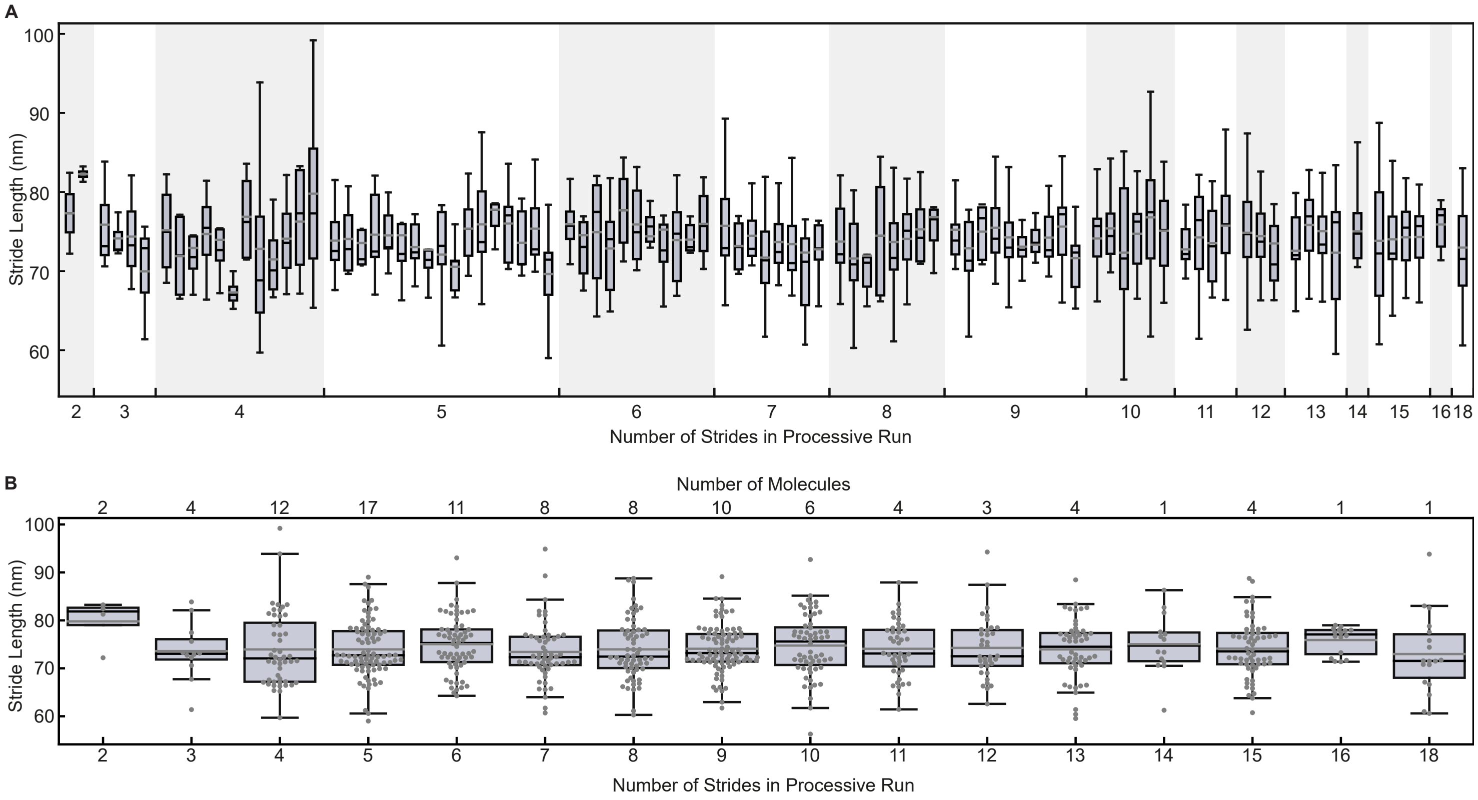

Figure S2. Characteristics of myosin-5a processive runs. Related to Figure 2. (A) Distribution of stride lengths for all tracked myosin-5a molecules. Molecules have been grouped by the number of strides in the run. Box plots of lengths of strides in processive run with each box corresponding to a single myosin molecule (N = 96 molecules, 725 strides). Boxes show quartiles of the dataset with error bars extending 1.5 times the inter-quartile range. Median and mean stride lengths marked in horizontal black and grey lines respectively. (B) Stride length is independent of processive run length. Data from each group in (A) have been combined. Grey dots mark every individual stride. Boxes show quartiles of each dataset with error bars extending 1.5 times the inter quartile range. Median and mean stride lengths marked in horizontal black and grey lines respectively.

bioRxiv preprint doi: https://doi.org/10.1101/2023.07.16.549178; this version posted July 16, 2023. The copyright holder for this preprint (which was not certified by peer review) is the author/funder, who has granted bioRxiv a license to display the preprint in perpetuity. It is made available under aCC-BY 4.0 International license.

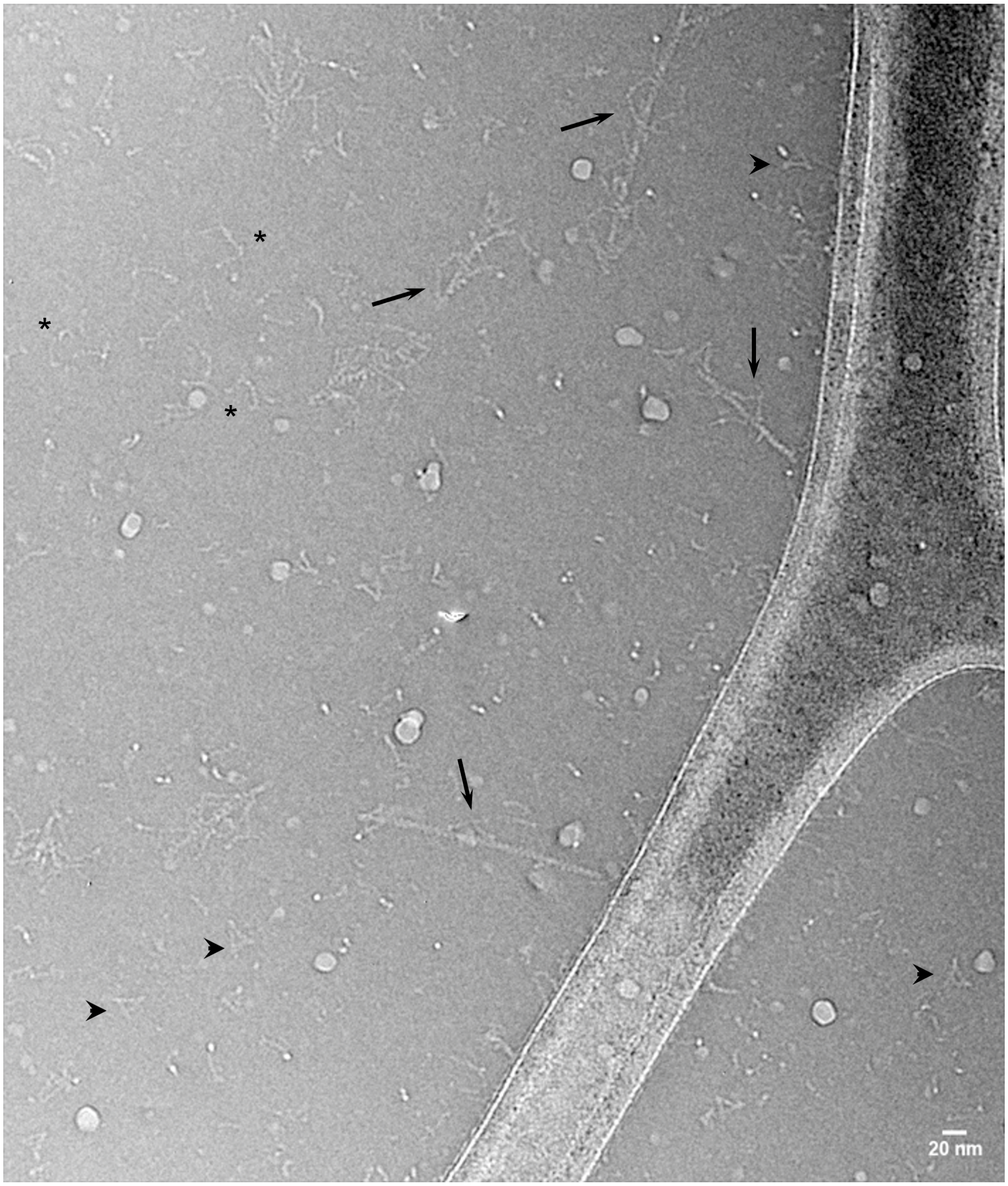

Figure S3. CryoEM field of full-length myosin-5a and F-actin in the presence of ATP. Related to Figure 3. Examples of extended myosin molecules bound by both heads to F-actin (arrows). Also compact molecules (arrowheads) and extended, detached molecules (asterisks). Contrast inverted (protein white). Scale bar: 20 nm.

bioRxiv preprint doi: https://doi.org/10.1101/2023.07.16.549178; this version posted July 16, 2023. The copyright holder for this preprint (which was not certified by peer review) is the author/funder, who has granted bioRxiv a license to display the preprint in perpetuity. It is made available under aCC-BY 4.0 International license.

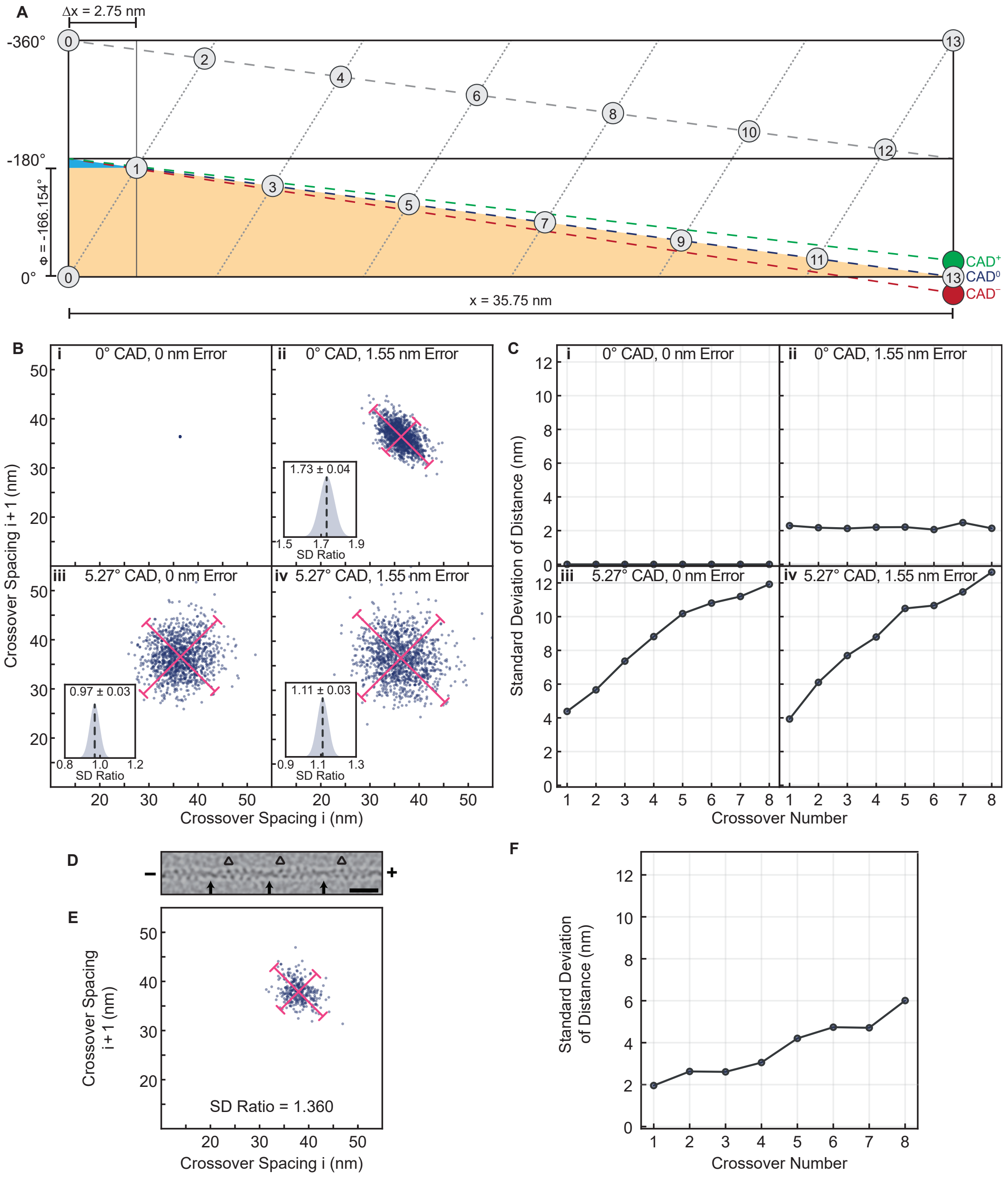

Figure S4. Analysis of actin filament CAD from cryoEM images. (A) Helical net representation of F-actin for deducing the relationship between rotation per actin subunit and crossover spacing. The net can be thought of as being made by dropping a hollow cylinder around an actin filament, marking the positions of the subunits on it, then cutting the cylinder lengthways and opening it out flat. The single, short-pitch, left-handed helix that passes through all the subunits is marked with short dashes; the two long-pitch, right-handed helices are marked by long dashes. Each crossover is where the long pitch helices cross the 0° and -180° lines. The net shown corresponds to the classical F-actin 13/6 helical geometry (13 subunits in 6 turns of the short-pitch helix), but the concept is general. The rotation per subunit (ϕ) of -166.154°, the rise per subunit (Δx) of 2.75 nm, and the crossover spacing (x) of 35.75 nm are all shown. CAD that produces a net decrease of subunit rotation over the 13 subunits (CAD^{-}) is shown by the red dashed line, which can be seen to move crossover position to the left, shortening crossover spacing. CAD^{+} (green dashed line) is the complementary phenomenon. Note the two similar triangles: the larger (orange) with apexes at 0°, -180°, actin subunit 13 and the smaller (blue) with apexes at -166.154°, -180°, actin subunit 1. (B) Scatter plots of successive actin crossover spacings in simulated actin filaments (100 subunits, 1,000 filaments) with and without CAD and Gaussian error in picking crossovers. Error bars denote 1.96 * the standard deviations along lines of slope -1 and +1. Insets: Gaussian distributions of the spread in the ratio of the standard deviations along the lines of slope -1 and +1, generated from 100 repeats. (C) The standard deviation of sequential crossover distances in 1,000 filaments, under the same conditions as in (B). (D) CryoEM image of F-actin[1], bandpass filtered to enhance subunit detail. Arrows mark position of crossovers. Triangles mark the position of triangles formed by three bold actin subunits on the barbed (+) side of each crossover that allows assignment of filament polarity. Scale bar: 20 nm. (E) Scatter plot of crossover spacing (i) against the neighboring crossover spacing towards the barbed end (i+1). (F) Measurement of the spread (SD) of values among a set of actin filaments, of the distance along the actin of a series of 8 consecutive crossovers away from a starting crossover.

bioRxiv preprint doi: https://doi.org/10.1101/2023.07.16.549178; this version posted July 16, 2023. The copyright holder for this preprint (which was not certified by peer review) is the author/funder, who has granted bioRxiv a license to display the preprint in perpetuity. It is made available under aCC-BY 4.0 International license.

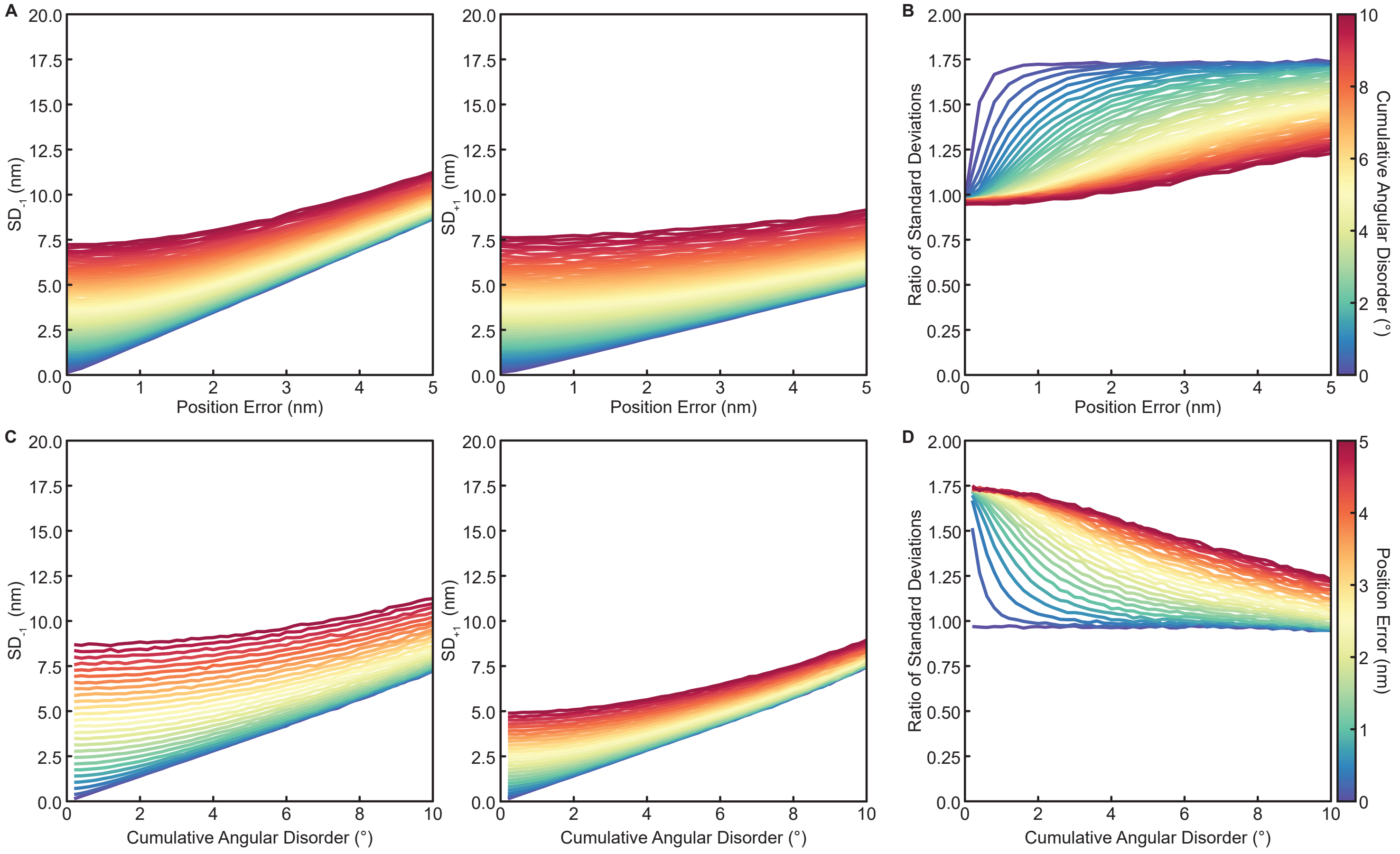

Figure S5. Actin filament CAD simulation of consecutive paired crossover distance. Related to Figure S4. (A) Standard deviation of simulated consecutive pair crossover scatter plots (1000 subunits, 1000 filaments) along lines of slope -1 and +1 (SD$_{-1}$, SD$_{+1}$) as a function of error from picking crossovers in determining the actin crossover spacings. The hue scales with magnitude of CAD. (B) The ratio SD$_{-1}$/ SD$_{+1}$ as a function of picking error. (C) and (D) The same simulated dataset transposed to show CAD along the x-axis and error from picking scaling as hue.

bioRxiv preprint doi: https://doi.org/10.1101/2023.07.16.549178; this version posted July 16, 2023. The copyright holder for this preprint (which was not certified by peer review) is the author/funder, who has granted bioRxiv a license to display the preprint in perpetuity. It is made available under aCC-BY 4.0 International license.

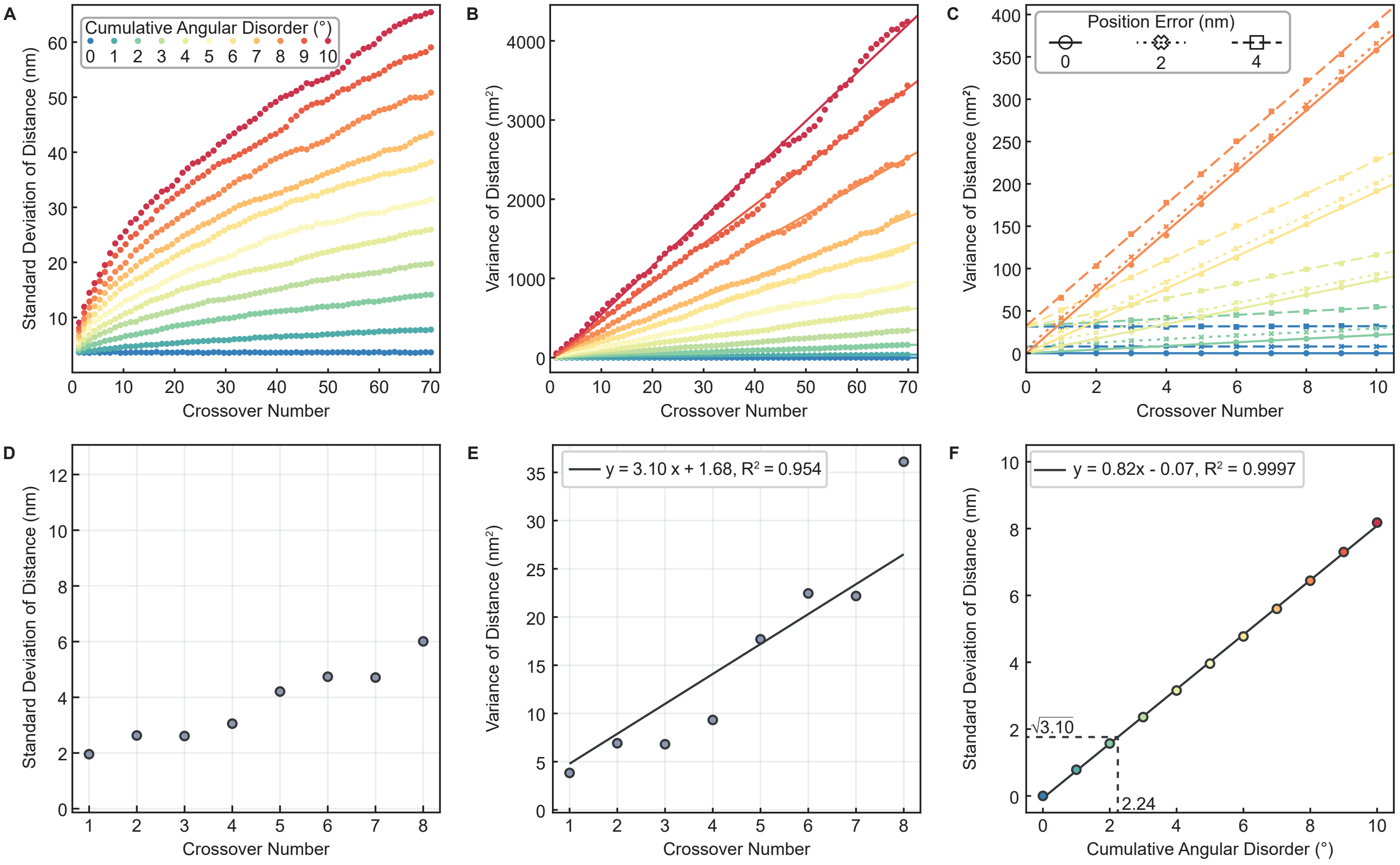

Figure S6. Actin filament CAD simulation of sequential crossover distance. Related to Figure S4. (A) Simulated standard deviations and (B) variances of sequential crossover distances extended to 70 crossovers per filament. The hue scales with cumulative angular disorder (CAD). Linear fits to the variances all have $r^2 \geq 0.992$. (C) Variation of gradient with picking error (10000 filaments). There is minimal change to the gradient with position errors of 0 nm (circles), 2 nm (crosses), and 4 nm (squares). Note that the offset caused by position error is given by 2(position error)^2 (so is 8 nm^2 and 32 nm^2, respectively), and can be measured as the intercept on the variance axis. (D) Standard deviation plot and (E) variance plot for the cryo-EM data from F-actin[1] Variance plot has line resulting from least squares fitting to estimate both CAD and measurement error (F) Calculated standard deviation of the first crossover as a function of CAD. A linear fit (R^2 = 0.9997) is shown. Interpolation of the cryoEM data at a gradient value of $\sqrt{3.10}$ as determined in (E) returns a CAD of 2.24 $\pm$ 0.05° for the cryo-EM data.

bioRxiv preprint doi: https://doi.org/10.1101/2023.07.16.549178; this version posted July 16, 2023. The copyright holder for this preprint (which was not certified by peer review) is the author/funder, who has granted bioRxiv a license to display the preprint in perpetuity. It is made available under aCC-BY 4.0 International license.

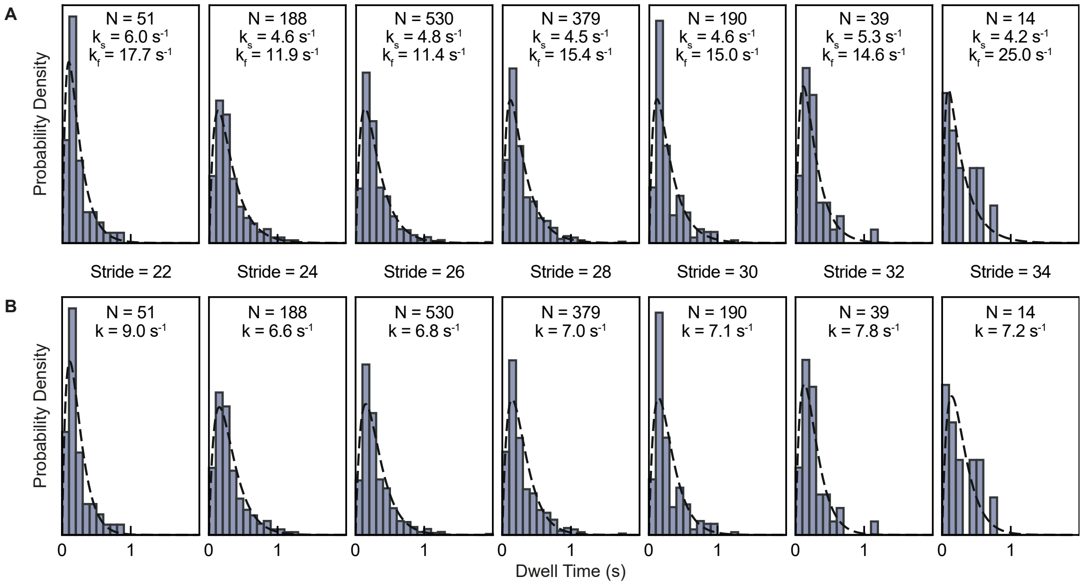

C

Step Length (actin subunits (asu))	Dwell Time Constant (s^{-1}) k_s	k_f	k
22	6.02 ± 1.21	17.66 ± 7.88	8.98 ± 0.89
24	4.58 ± 0.55	11.93 ± 3.06	6.62 ± 0.34
26	4.83 ± 0.36	11.36 ± 1.67	6.78 ± 0.21
28	4.55 ± 0.32	15.35 ± 2.65	7.02 ± 0.25
30	4.64 ± 0.44	14.99 ± 3.19	7.09 ± 0.36
32	5.33 ± 1.23	14.62 ± 7.06	7.81 ± 0.88
34	4.19 ± 1.32	24.99 ± 25.40	7.18 ± 1.36

Figure S7. Fitting of trailing head dwell times to a two-step reaction sequence. Related to Figure 6. Panels show dwell time distributions (bars) and results of a global maximum likelihood fitting (dashed lines) to a convolution of two exponentials. (A) Rate constants assumed to be different. k_s is set to be lower than k_f and can therefore correspond to either the rate constant for ADP release or of the subsequent ATP binding step. (B) Rate constants assumed to be the same. (C) Dwell time rate constants with error estimates are tabulated from a global maximum likelihood fitting of the dwell time data to a convolution of two exponentials when the two rate constants are allowed to differ and when they are constrained to be the same.
